# Proteome Analysis Reveals Extensive Light Stress-Response Reprogramming in the Seagrass *Zostera muelleri* (Alismatales, Zosteraceae) Metabolism

**DOI:** 10.3389/fpls.2016.02023

**Published:** 2017-01-17

**Authors:** Manoj Kumar, Matthew P. Padula, Peter Davey, Mathieu Pernice, Zhijian Jiang, Gaurav Sablok, Loretto Contreras-Porcia, Peter J. Ralph

**Affiliations:** ^1^Climate Change Cluster, Faculty of Science, University of Technology Sydney (UTS)Sydney, NSW, Australia; ^2^School of Life Sciences and Proteomics Core Facility, Faculty of Science, University of Technology Sydney (UTS)Sydney, NSW, Australia; ^3^Key Laboratory of Tropical Marine Bio-resources and Ecology, South China Sea Institute of Oceanology, Chinese Academy of Sciences (CAS)Guangzhou, China; ^4^Departamento de Ecología y Biodiversidad, Facultad de Ecología y Recursos Naturales, Universidad Andres BelloSantiago, Chile; ^5^Center of Applied Ecology and Sustainability (CAPES), Facultad de Ciencias Biológicas, Pontificia Universidad Católica de ChileSantiago, Chile

**Keywords:** *Zostera muelleri*, light stress, proteome profiling, 2D-IEF, seagrass

## Abstract

Seagrasses are marine ecosystem engineers that are currently declining in abundance at an alarming rate due to both natural and anthropogenic disturbances in ecological niches. Despite reports on the morphological and physiological adaptations of seagrasses to extreme environments, little is known of the molecular mechanisms underlying photo-acclimation, and/or tolerance in these marine plants. This study applies the two-dimensional isoelectric focusing (2D-IEF) proteomics approach to identify photo-acclimation/tolerance proteins in the marine seagrass *Zostera muelleri*. For this, *Z. muelleri* was exposed for 10 days in laboratory mesocosms to saturating (control, 200 μmol photons m^−2^ s^−1^), super-saturating (SSL, 600 μmol photons m^−2^ s^−1^), and limited light (LL, 20 μmol photons m^−2^ s^−1^) irradiance conditions. Using LC-MS/MS analysis, 93 and 40 protein spots were differentially regulated under SSL and LL conditions, respectively, when compared to the control. In contrast to the LL condition, *Z. muelleri* robustly tolerated super-saturation light than control conditions, evidenced by their higher relative maximum electron transport rate and minimum saturating irradiance values. Proteomic analyses revealed up-regulation and/or appearances of proteins belonging to the Calvin-Benson and Krebs cycle, glycolysis, the glycine cleavage system of photorespiration, and the antioxidant system. These proteins, together with those from the inter-connected glutamate-proline-GABA pathway, shaped *Z. muelleri* photosynthesis and growth under SSL conditions. In contrast, the LL condition negatively impacted the metabolic activities of *Z. muelleri* by down-regulating key metabolic enzymes for photosynthesis and the metabolism of carbohydrates and amino acids, which is consistent with the observation with lower photosynthetic performance under LL condition. This study provides novel insights into the underlying molecular photo-acclimation mechanisms in *Z. muelleri*, in addition to identifying protein-based biomarkers that could be used as early indicators to detect acute/chronic light stress in seagrasses to monitor seagrass health.

## Introduction

Seagrasses are marine ecological engineers and are rated the third most valuable ecosystem globally with the average global value for their ecological services estimated at US $28,916 ha^−1^years^−1^ (Costanza et al., 2014). However, they are declining at an alarming rate (by >7% years ^−1^) as a result of climate change and human activities (Waycott et al., 2009), attributed directly (e.g., dredging), or indirectly (e.g., eutrophication) to light stress (York et al., 2013).

Seagrasses are monocotyledonous flowering plants, which have experienced extreme evolutionary events in the angiosperm lineage before adapting to the marine habitat 130 million years ago (Olsen et al., 2016). Greater than 70 species of seagrasses have been identified, growing submerged, and rooted in soft bottom estuarine and marine environments around the world except in Antarctica (Coles et al., 2015). Seagrass species from the genus *Zostera* are among the most important and widely distributed species. They are considered model organisms for ecological, demographic and genetic studies (Golicz et al., 2015).

Light availability is considered the most important determinant for seagrass productivity, distribution, and abundance. Seagrasses have unusually high light requirements for growth (10–37% of surface irradiance compared with 0.11% for most other marine macrophytes), which make them highly vulnerable to deterioration in water clarity (Petrou et al., 2013; Chartrand et al., 2016). In coastal habitats, increased light scattering, and/or light attenuation due to suspended particles or by the overgrowth of epiphytes or algal blooms in the water column affects light quality. Moreover, seagrasses growing in intertidal and shallow aquatic environments are regularly exposed to super-saturating irradiance for part of the day, and even to full sunlight, which can lead to light stress. Seagrasses are also often exposed to highly fluctuating light fluxes due to waves (focusing) and tidal movement (Schubert et al., 2015). Taking into account these environmental fluctuations, seagrass require physiological, and morphological adaptations to withstand a wide range of light stress. Tolerance to light stress often varies among seagrass species (Orth et al., 2006; Petrou et al., 2013; Collier et al., 2016). Therefore, new knowledge on the light thresholds and the cellular mechanisms for seagrass survival under light stress can inform better management of seagrass habitats.

Over the past decade, seagrass research has been centered on understanding the effect of light limitation on photosynthetic performance and growth (Silva et al., 2013; York et al., 2013; Dattolo et al., 2014; Park et al., 2016). At physiological levels, seagrasses under light limitation exhibit a general increase in the slope of photosynthesis-irradiance curve (α) and a lower light saturation point (Ek) to enhance their light harvesting efficiency by increasing photosynthetic pigments pool and antenna size (Ralph et al., 2002, 2007; Zimmerman, 2006; Howarth and Durako, 2013; Silva et al., 2013; Dattolo et al., 2014; Park et al., 2016). Further, involvement of xanthophyll cycle in the dissipation of excess energy and accumulation of anthocyanin pigments has been suggested to prevent photosystem from photodamage during high light conditions in various seagrasses (Novak and Short, 2011; Howarth and Durako, 2013; Marín-Guirao et al., 2013). However, studies to date have provided evidence that the limits of light deprivation are seagrass specific. For example, *Zostera marina* is less resilient to light reduction than *Cymodocea nodosa*, mostly due to less plasticity in its pigment pools and reduced efficiency for carbohydrate storage and usage during shading (Silva et al., 2013). Similarly, low resilience to shading was also observed with *Z*. *muelleri*, the leaf and shoot density being reduced together with more leaf senescence and less storage capacity under light limitation (Collier et al., 2012; York et al., 2013). More recently, the individual variability of *Z. marina* response to shading stress has also been reported with the differential regulation of genes involved in carbohydrate metabolism and photosynthesis (Salo et al., 2015). In contrast to light limitation, the response of seagrasses to high light has received less attention.

Resilience of seagrasses to light fluctuations depends on their ability to either tolerate or acclimate to light, by reprogramming their cellular machinery at the gene, protein and metabolite levels. The studies of transcriptional fluxes have provided insight into gene-specific behavior. However, from the point of translational protein level, it is yet to be ascertained whether regulation at the transcriptional level is a consequence of light limitations or is just a conditional bias. Since, proteins respond dynamically to environmental fluctuations, proteomics can provide novel insights into the cellular pathways and biochemistry of stress tolerance mechanism to environmental clues. Performing differential displays of the proteome in land plants under contrasting conditions is now a common method (see review, Ghosh and Xu, 2014; Komatsu et al., 2014; Kosová et al., 2014). However, only few seagrasses species (*Posidonia oceanica* and *Cymodocea nodosa*) have been examined using this novel technology (Mazzuca et al., 2009; Dattolo et al., 2013; Piro et al., 2015) to address their response to light and salinity stress, respectively.

The underlying cellular tolerance and/or acclimation mechanisms to low and high light regimes in seagrass remain elusive at the proteome level in the *Zostera* genus. In Australia, *Z. muelleri* (syn. *Z. capricorni*) is a dominant seagrass widely distributed in intertidal zones of temperate and tropical shallow waters ranging from the south and east coasts (Golicz et al., 2015). This study aims to gain insights into the acclimation response of *Z. muelleri* to light induced stress in laboratory mesocosms. Therefore, a comparative analysis using Two-dimensional Gel Electrophoresis (2-DE) was performed to reveal the protein profile in leaves exposed to low and high light intensities to identify the differentially abundant proteins involved in light stress acclimation. This study identifies protein-based biomarkers that can be used as early indicators for detecting acute/chronic light stress in seagrasses.

## Materials and methods

### Collection and maintenance of seagrass and light exposure treatments

Samples of *Z. muelleri* were harvested from Narrabeen Lagoon (34°11′75″South, 62°68′134″East), Sydney, NSW, Australia. Environmental variables including temperature (22°C), salinity (28 ± 1 S_A_) and irradiance were measured at seagrass canopy height, so that the natural environment of Narrabeen Lagoon could be replicated in the Seagrass Mesocosms Facility in the Climate Change Cluster at UTS. Irradiance was measured using a hand-held meter, with attached underwater 2-π downward irradiance sensor (LiCor 250A, Nebraska, USA). Rapid Light Curves (RLCs) were also conducted on seagrass plants at the collection site to determine suitable irradiance treatments in the laboratory by using a Diving-Pulse Amplitude Modulated fluorimeter (DIVING-PAM, Heinz Walz GmbH, Eichenring, Germany; Supplementary Table 1). RLCs measurements at samples collection site indicated that photosynthetic saturating light was approximately 200 μmol photons m^−2^ s^−1^. Turfs of seagrass with 10–15 cm of intact sediment were carefully removed from the meadow using a hand spade and placed in plastic tubs. Wet paper towels were placed over the plants to prevent desiccation during transport to the laboratory within 1 h under cool conditions.

In the laboratory, plants were cleaned of epiphytes and grazers. Additionally, any intact sediment was washed from roots and rhizomes using saline water 27S_A_ [S_A_ is absolute salinity (i.e., mass fraction of salt in sea water, a newly introduced standard to measure salinity; Wright et al., 2011; http://www.teos-10.org)]. Individual shoots were then separated at the horizontal rhizome, approximately 40 individual shoots were planted about 2 cm into the sediment (approximately 40% natural sediment, 60% washed sand) in individual aquaria (50 L, equipped with pump and diffusive airstone). Three aquaria replicates were used for each of the three light treatments: the control, saturating light (SL; 200 μmol photons m^−2^ s^−1^), supersaturating light (SSL; 600 μmol photons m^−2^ s^−1^), and limited light (LL; 20 μmol photons m^−2^ s^−1^) irradiance. Aquaria were then filled with fresh seawater (27 S_A_), and the temperature was set to 22°C in order to replicate the conditions of Narrabeen Lagoon. Pump velocity and air-stone flow rate were kept the same across all aquaria to ensure effective stirring of the water body and gaseous diffusion.

LED lights were suspended above each aquarium (Cidley 250W; 4 channel; red, blue, white, and green), and utilizing field data, all lights were programmed to provide a daily sinusoidal regime. For acclimation, all aquaria were subjected to sinusoidal light regimes for a fortnight, with a mid-day maximum irradiance of 200 μmol photons m^−2^s^−1^ of light. The sinusoidal regime consisted of 11 h of ramping light and 13 h of constant darkness, based on proximate sunset and sunrise times. On the final night of acclimation, when lights entered the dark stage (to minimize disruption) in limited light, and super-saturating light treatment tank, lights were re-programmed to pre-determined sinusoidal treatment regimes with a maximum midday irradiance of 20 μmol photons m^−2^ s^−1^ (LL) and 600 μmol photons m^−2^ s^−1^ (SSL). After 10 days of experimental treatments, plants were collected (whole plant leaves only) in three biological replicates from light treatments including controls. The samples were snap frozen in liquid N_2_ before being stored at −80°C for proteomic analysis.

### Fluorescence measurements

To determine the ability of *Z. muelleri* to adjust and acclimatize to SSL and LL regimes, a Diving PAM (Walz GmbH, Effeltrich, Germany), Diving F-probe and leaf clip was used, focusing on the area at approximately 2–3 cm above the leaf sheath of the second leaf (Ralph and Gademann, 2005). Rapid light curves (RLCs) were performed using a Diving PAM with the following settings: measuring intensity (8), saturation width (0.8 s), gain (4), light curve width (0:10), and light curve intensity (1). The following 8 actinic light levels (μmol photons m^−2^ s^−1^) were used 0, 13, 51, 106, 182, 268, 363, 528, and 722. Before initiation of the experiment, RLCs were conducted to ensure acclimation was successful and no significant variation was present across treatments (Supplementary Figure 1A). RLCs were also taken at the end point of the experiment, Day 10 (Supplementary Figure 1B). Identical settings were utilized for Diving PAM as observed in the field across all treatments. All data was downloaded from the Diving PAM via WinControl 3.0 and exported to Microsoft Excel and Sigmaplot V 12.5, wherein RLCs were plotted according to Ralph and Gademann (2005). Relative Maximum Electron Transport Rate (ETR_max_) and minimum saturating irradiance (Ek) values were then derived from curves.

### Protein extraction and purification

Proteins were extracted from whole leaves according to Wang et al. (2007) by pulverizing the leaf tissue using a cryomill (Retsch MM200) with a 1 cm stainless steel ball. Extracted proteins were precipitated with 100 mM ammonium acetate in methanol overnight at −20°C. The precipitated proteins were solubilized in rehydration buffer containing 7 M Urea, 2 M Thiourea, 0.5% C7BzO (UTC7) and 50 mM Tris-HCl pH 8.8, followed by the reduction and alkylation of disulfide bonds in a single step, for 90 min at room temperature, using the reducing agent tributylphosphine (TBP, 5 mM) and an alkylating acrylamide monomer (AM, 20 mM). The reaction was quenched using dithiothreitol (DTT, 20 mM). Protein samples were desalted using MicroBioSpin (Bio-Rad) equilibrated with UTC7 according to the manufacturer's instructions. Protein concentration was determined by SDS-PAGE and densitometry using bovine serum albumin as the standard.

### Two-dimensional electrophoresis (2-DE), gel scanning, and image analysis

Protein (300 μg) was analyzed using iso-electric focusing (IEF). A cup-loading method was used. Immobilized pH gradient (IPG) strips (Bio-Rad, pH 3–10, 11 cm) were passively rehydrated with rehydration solution (UTC7) for a minimum of 6 h at room temperature. Isoelectric focusing was conducted in a Protean IEF device (Bio-Rad). After isoelectric focusing, gel strips were equilibrated in equilibration buffer, prior to SDS-PAGE in the second dimension. Gels were then fixed with 40% methanol and 10% acetic acid for 30 min prior to protein staining with Coomassie Stain G-250. Gels were then destained and imaged using a fluorescence scanner (Typhoon FLA-3500). Molecular masses were estimated using a broad-range standard (Precision Plus, Bio-Rad) that co-migrated in the SDS-PAGE gel. Gel images were analyzed using PDQuest software (Version 8.0; BioRad, USA). Spot densities were expressed as mean normalized volumes and fold changes between light treatments and control samples were calculated. Based on the program's statistical analysis (One-Way Analysis of Variance), spots with a *p* < 0.05 and a fold change of ≥1.5 were selected for subsequent identification using tandem mass spectrometry. A total of nine 2-DE gels originating from three individual replicates of each light treatment were analyzed. The terms up- and down- regulated (UR, DR) were used to describe differentially regulated proteins in (i) SSL samples compared to Control samples, and in (ii) LL samples compared to Control samples. Protein spots that were detected in LL and/or SSL treatments, but not in Control samples, were termed “newly appeared (NA).”

### Protein identification and bioinformatics analysis

Selected differentially regulated protein spots were excised from gels, trypsin digested, and analyzed by LC/MS/MS according to Pokharel et al. (2016). Using an autosampler, connected to a nanoLC system (Tempo Eksigent, USA), 10 μL of the sample was loaded at 20 μL/min with MS loading solvent (2% Acetonitrile + 0.2% Trifluoroacetic Acid) onto a C8 trap column (CapTrap. Michrom Biosciences, USA). After washing the trap for three min, the peptides were washed off the trap at 300 nL/min onto a PicoFrit column (75 μm × 100 mm) packed with Magic C18AQ resin (Michrom Biosciences, USA). Peptides were eluted from the column and into the source of a QSTAR Elite hybrid Quadrupole-Time-of-Flight mass spectrometer (Applied Biosystems/MDS Sciex) using the following program: 5–50% MS solvent B (98% Acetonitrile + 0.2% Formic Acid) over 8 min, 50–80% MS buffer B over 5 min, 80% MS buffer B for 2 min, 80–5% for 3 min. MS solvent A consisted of 2% Acetonitrile + 0.2% Formic Acid. The eluting peptides were ionized with a 75 μm ID emitter tip that tapered to 15 μm (New Objective) at 2300 V. An Intelligent Data Acquisition (IDA) experiment was performed, with a mass range of 375–1500 Da continuously scanned for peptides of charge state 2+–5+ with an intensity of more than 30 counts/s. Selected peptides were fragmented and the product ion fragment masses measured over a mass range of 100–1500 Da. The mass of the precursor peptide was then excluded for 15 s.

Peptides were identified and protein identity inferred using both Mascot and PEAKS Studio software (Peaks Studio 7.5, Bioinformatics Solutions Inc., Waterloo, ON, Canada). The MS/MS data files produced by the QSTAR were searched using the software Mascot Daemon (version 2.4) and searched against the LudwigNR database (comprised of the UniProt, plasmoDB, and Ensembl databases (vQ111. 16,818,973 sequences; 5,891,363,821 residues). The settings used were as follows—Fixed Modifications: none; Variable Modifications: carbamidomethyl, propionamide, oxidized methionine; Enzyme: semi-trypsin; Number of Allowed Missed Cleavages: 3; Peptide Mass Tolerance: 100 ppm; MS/MS Mass Tolerance: 0.2 Da; Charge State: 2+ and 3+.

The results of the search were then filtered by including only protein hits with at least one unique peptide (Bold Red) and excluding peptide hits with a *p* > 0.05. Peptides identified by Mascot were further validated by manual inspection of the MS/MS spectra for the peptide to ensure the b- and y-ion series were sufficiently extensive for an accurate identification. For further protein identification, the Uniprot database of *Z. marina* and the customized database generated by converting ESTs of different seagrasses into protein sequences, were integrated into the Mascot database, and searched using PEAKS Studio v7.5 using the same parameters as Mascot. Later, the PEAKS studio search results were exported into a DAT FILE and normalized and quantified using Scaffold Version 4.0 software. The threshold selection for the protein sequences was a PEAKS protein score >20 (the sum of the supporting peptide scores for each distinct sequence that are a representation of the *p*-value in PEAKS as a proxy of the LDF score, which measures the quality of the peptide-spectrum match; López-Cristoffanini et al., 2015). Only proteins showing at least one peptide with an individual score confidence >20 in PEAKS, when the scaffold parameter was set at a protein threshold of 90% and peptide threshold of 95%, were considered as valid candidates. For these proteins, MS/MS spectra were also manually validated by the presence of a series of at least four *y*-ions.

After PEAKS identification, protein sequences were analyzed using BLAST-P to determine similarity with known proteins in the NCBI database. The threshold was set to a minimal significance of 1e−3 and an identity percentage of >25%. The theoretical p*I* and molecular weight of the blast hit was calculated using the ExPASy tool (http://web.expasy.org/compute_pi/). The identified proteins were assigned to Gene Ontology (GO) using Blast2GO software (https://www.blast2go.com/). The protein pathway analysis was performed using KOBAS 2.0 (http://kobas.cbi.pku.edu.cn). The GO database, BLAST annotations and information reported in the literature were used to categorize each of the identified proteins. Subcellular localization of the proteins was Plant-mLoc (http://www.csbio.sjtu.edu.cn/bioinf/plant-multi/) using accessions of *Z. marina* (complete genome sequence available), phylogenetically close to *Z. muelleri*.

### Statistical analysis

Statistical analysis was conducted using IBM-SPSS Statistics 19 software. Shapiro-Wilk and Levene's tests confirmed the normality and homoscedasticity of the data, respectively. For fluorescence measurements, Two-Way ANOVA was performed using *post hoc* simple main affect univariate analysis, and the values were represented as the mean of four biological replicates with standard deviation. For proteomic analysis, One-Way ANOVA followed by Tukey's multiple comparisons tests (T) was performed considering treatments as the fixed factor (control and super-saturating light/limited light, using three biological replicates for each condition). Differences between mean values were considered to be significant at a probability of 5% (*p* < 0.05) for both fluorescence and proteomic analysis. The size effect of each condition in proteomic analysis was also determined estimating Cohen's *d* absolute value according to López-Cristoffanini et al. (2015).

## Results

### Photosynthetic performance

**Figure 1:**
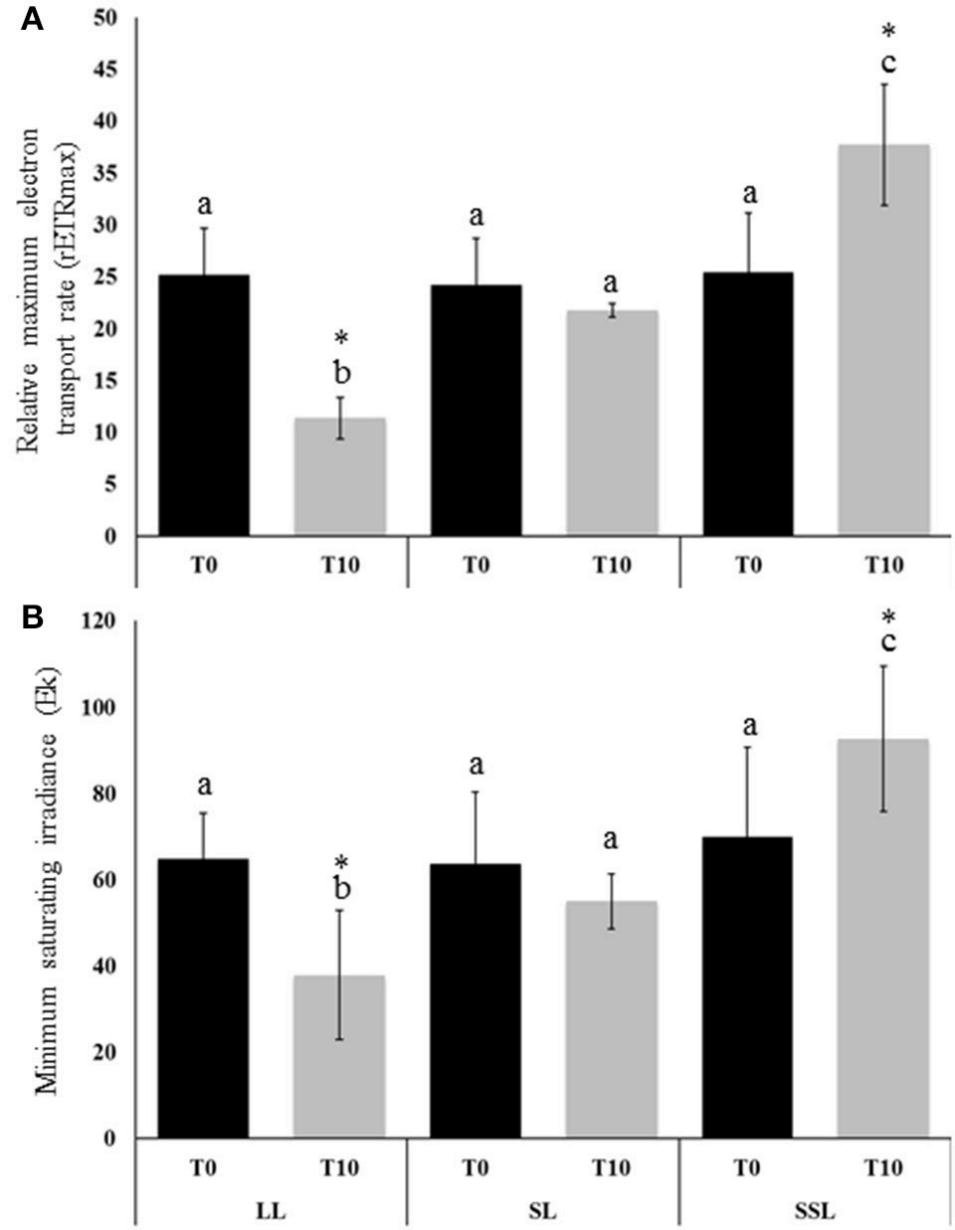
**Relative Maximum Electron Transport Rate (rETRmax) and minimum saturating irradiance (Ek) for *Zostera muelleri* seagrass plants exposed to saturating (SL, Control; 200 μmol photons m^−2^s^−1^), super-saturating light (SSL; 600 μmol photons m^−2^s^−1^), and limited light (LL; 20 μmol photons m^−2^s^−1^) irradiance at (A)** day 0 (T0; black column) and **(B)** day 10 (T10; gray column) of the experiment. Different letters on the similar shade columns indicate mean values for a particular day that were significantly differed at (*p* ≤ 0.05). (^*^) on the different shade columns indicate significant differences in the mean values for the interaction between time and treatments at (*p* ≤ 0.05) (Mean ± S.D, *n* = 4) analyzed by Two-Way ANOVA.

To assess the effect of SSL and LL treatments on photosynthetic performance of the seagrass *Z. muelleri*, relative maximum ETR (rETR_max_) was measured in the second leaf at the start (0 day, T0) and at the end of the experiment (10 days, T10). The mean values of rETR_max_ at Control, SSL and LL conditions at T0 were recorded as 24.16 ± 4.51, 25.21 ± 4.44 and 25.43 ± 5.72, respectively (Figure 1). No significant difference in rETR_max_ values at T0 suggests that the experiment was initiated when all plants were acclimatized to similar conditions in the indoor laboratory mesocosms. However, a significant change in the rETR_max_ values at T10 was observed for seagrass plants exposed to different light conditions with corresponding values as 11.36 ± 1.99 (LL), 21.75 ± 0.63 (Control), and 37.71 ± 5.86 (SSL), respectively (Figure 1A). The difference in rETR_max_ at T10 was also significant for the interaction between time and treatments (Two-Way ANOVA; time: F1, 18 = 0.569, *p* = 0.46; treatment: F2, 18 = 19.54, *p* < 0.0005; interaction: F2, 18 = 18.456, *p* < 0.0005). With respect to Ek, there was no significant difference at T0 between different light conditions (determined by *post-hoc* simple main affect univariate analysis; *p* = 0.824) with mean values 64.83 ± 10.64 (LL), 63.77 ± 16.46 (SL), and 69.99 ± 20.65 (SSL) (Figure 1). However, at T10 Ek differed significantly with corresponding mean values as 37.92 ± 14.919 (LL), 55.03 ± 6.333 (Control), and 92.60 ± 16.819 (SSL) (Figure 1B). For Y(I), no significant differences or interactions were observed across the duration of the entire experiment (data not shown).

### Proteomic profiling

To study the molecular mechanism of photo-acclimation of the *Z. muelleri* to different light conditions, proteome profiles were compared at T10. At the proteome level, the results indicated that the differences observed in rETR_max_ at T10 affected the protein profile. After 2-DE separation and Coomassie Blue staining, the average numbers of spots detected on gels were 389, 476, and 332, for control (SL), SSL and LL conditions, respectively. Comparative analysis of the proteome visualized in 2-DE gels (*p*I 3–10) revealed that a total of 93 and 40 spots underwent changes in volume variation (1.5-fold, *p* < 0.05) under SSL and LL conditions respectively, compared to the Control.

**Figure 2:**
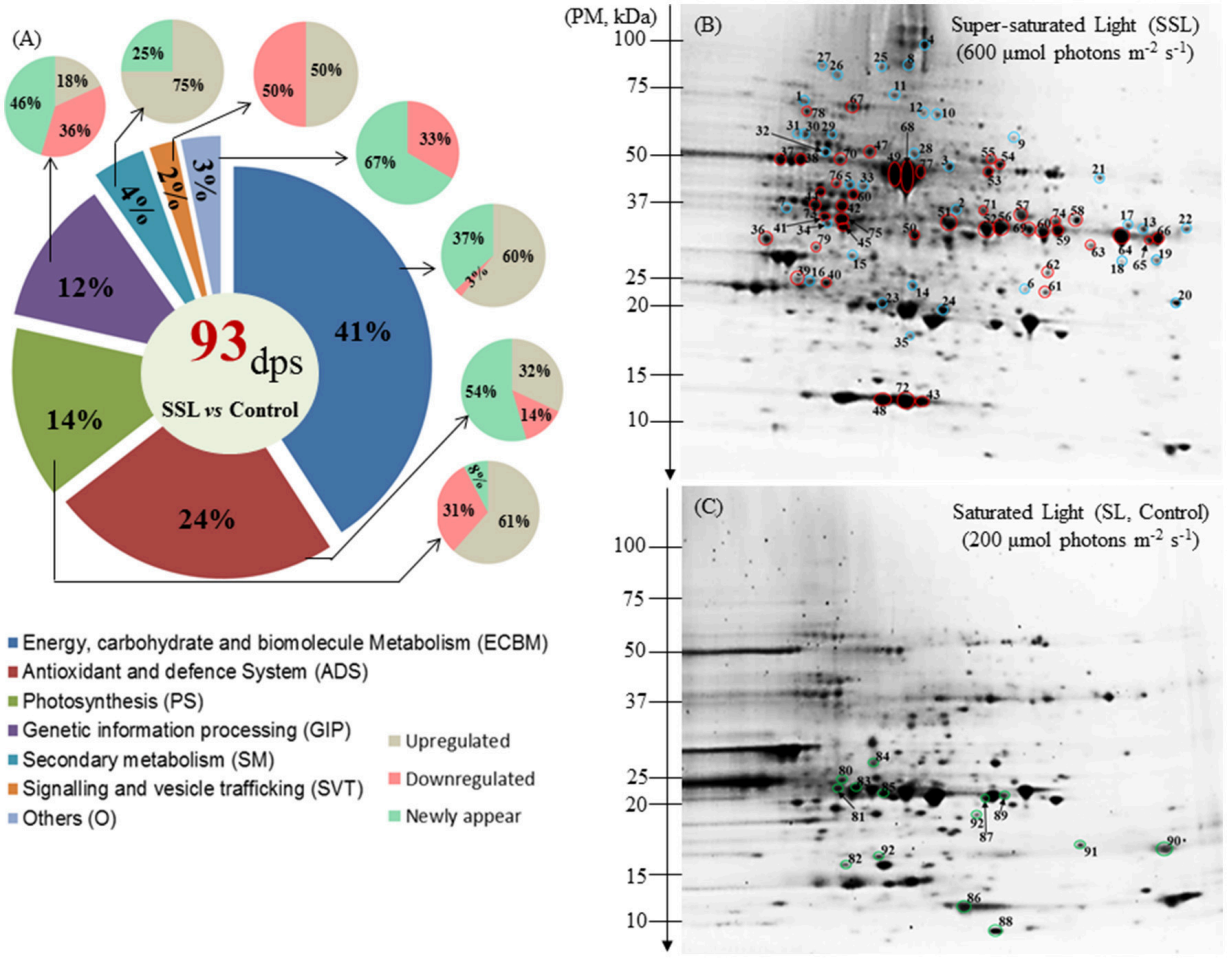
**Protein distribution and profiling of *Zostera muelleri* exposed to light stress. (A)** Functional group classification of differentially expressed proteins. **(B)** Representative 2-DE image (*p*I range 3–10, *n* = 3) of the leaf proteome exposed to super-saturating light (SSL; 600 μmol photons m^−2^s^−1^), **(C)** saturating light (SL, Control; 200 μmol photons m^−2^s^−1^) irradiance conditions. Spots circled in red, blue and green represent up-regulated, newly appeared and down-regulated proteins. Gels were stained with Coomassie Blue G-250. Dps, differential protein spots; PM, protein marker.

**Figure 3:**
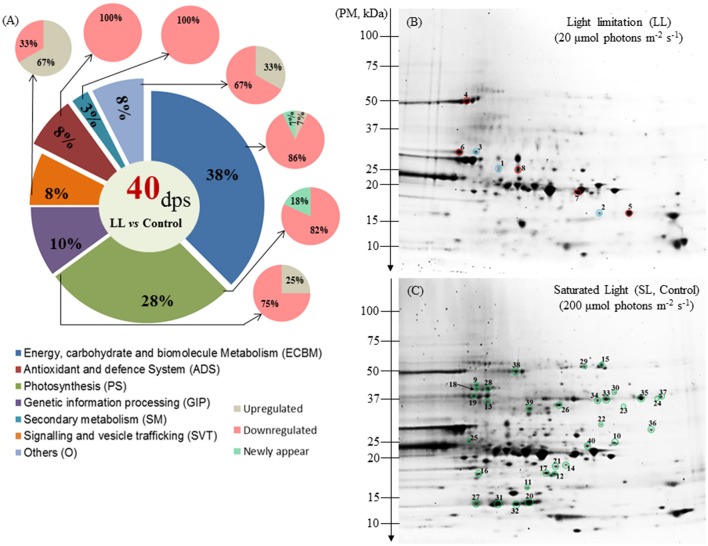
**Protein distribution and profiling of *Zostera muelleri* exposed to light stress. (A)** Functional group classification of differentially expressed proteins. **(B)** Representative 2-DE (pI range 3–10, *n* = 3) of the leaf proteome exposed to limited light (LL; 20 μmol photons m^−2^s^−1^), **(C)** saturating light (SL, Control; 200 μmol photons m^−2^s^−1^) irradiance conditions. Spots circled in red, blue and green represent up-regulated, newly appeared and down-regulated proteins. Gels were stained with Coomassie Blue G-250. Dps, differential protein spots; PM, protein marker.

All the differentially regulated spots were successfully characterized by LC-MS/MS and identified by bioinformatic analysis. The identified proteins were classified into the following seven categories according to their function: (1) energy, carbohydrate and biomolecules metabolism (ECBM); (2) photosynthesis (PS); (3) antioxidant and defense system function (ADS); (4) genetic information processing (GIP); (5) secondary metabolism (SM); (6) signaling and vesicle trafficking (SVT); and (7) others (Figures 2, 3).

#### Comparative proteome analysis of plants exposed to control and SSL conditions

Among the 93 spots that were differentially expressed under SSL conditions, compared to the Control light condition (SL), a total of 77 spots with defined accession IDs were successfully retrieved using the Batch Entrez NCBI database after filtering duplicate IDs (16). Most of the accessions were matched with *Z. marina* (97.6%), and others with *Z*. *noltei* (1.2%) and *Z*. *angustifolia* (1.2%).

Of the 93 differentially expressed protein spots, 44 were up-regulated (UR, 47%), 14 were down regulated (DR, 15%) and 35 were newly appeared (NA, 38%) (Table 1, Figures 2A–C). Within the seven categories, UR and DR proteins significantly varied between Control and SSL (*p* < 0.05). Cohen's *d* absolute values ranged from 2.86 to 17.58. The most sensitive cellular pathway responding in *Z*. *muelleri* exposed to SSL conditions was ECBM with 41% differential regulated proteins (UR 60%, DR 3%, NA 37%); followed by ADS with 24% (UR 32%, DR14%, NA 54%); PS 14% (UR 61%, DR 31%, NA 8%) and GIP 12% (UR 18%, DR 36%, NA 46%) (Figure 2A). Proteins belonging to functional category SM, SVT and others were least effected with 4, 2, and 3% of differentially regulated proteins (Figure 2A).

**Table 1:** **Identification of proteins with varied normalized volumes between saturating light (SL, Control, 200 μmol photons m^−2^ s^−1^) and super-saturating light irradiance (SSL, 600 μmol photons m^−2^ s^−1^) stress of *Zostera muelleri***.

**Protein**	**Spot no**.	Accession^*^	**Subcellular localization^a^**	**Regulation**	**pI**		**Mr (kD)**		**Score (PEAKS)**	**Pep^b^**	**Uni^c^**	**SC**
					**Obs**.	**Theo**.	**Obs**.	**Theo**.				
**ENERGY, CARBOHYDRATE, AND BIOMOLECULE METABOLISM**												
Aconitase	25	KMZ63341.1	MT	NA	6.19	6.04	89.12	106.13	263.14	32	10	41
Adenosylhomocysteinase	47	KMZ66813.1	PX	UR	5.98	5.6	59.77	53.58	173.51	14	14	31
Aminomethyltransferase	17	KMZ75789.1	MT/CY	NA	8.46	9.01	40.71	44.21	239.20	27	5	54
Aminomethyltransferase	22	KMZ75789.1	MT/CY	NA	9.02	9.01	40.94	44.21	234.22	28	7	61
Aspartate transaminase	58	KMZ73817.1	CL	UR	7.91	7.72	40.96	44.36	256.22	37	5	50
Aspartate transaminase	2	KMZ64674.1	MT	NA	6.71	6.51	42.55	47.74	243.47	27	6	66
ATP synthase beta subunit, P	37	AAK72873.1 ^#^	CL	UR	5.12	5.17	55.20	51.26	255.78	30	4	62
Citrate (Si)-synthase	9	KMZ62606.1	MT	NA	7.31	6.88	66.19	64.12	252.53	25	16	46
Dihydrolipoyl dehydrogenase	55	KMZ64155.1	MT	UR	7.06	6.72	57.50	53.57	227.64	27	19	55
F-ATPases	38	KMZ61829.1	CL/ML	UR	5.31	5.64	56.23	59.50	278.62	24	22	68
Fructose-bisphosphate aldolase	74	KMZ58915.1	CY	UR	7.72	7.54	39.92	38.60	247.17	35	10	76
Fructose-bisphosphate aldolase	13	KMZ58915.1	CY	NA	8.60	7.54	40.40	38.60	244.95	34	12	78
GA3P dehyrogenase (P)	60	KMZ61796.1	CL	UR	7.61	7.63	37.85	43.31	264.39	36	12	64
GA3P dehyrogenase (P)	56	KMZ64911.1	CY	UR	7.20	6.97	38.68	36.47	234.96	26	15	66
GA3P dehyrogenase (P)	59	KMZ61796.1	CL	UR	7.76	7.63	38.22	43.31	212.00	23	7	49
GA3P dehyrogenase (P)	64	KMZ61796.1	CL	UR	8.40	7.63	38.30	43.31	262.32	29	10	60
Glutamate decarboxylase	70	KMZ69611.1	MT/CL	UR	5.69	5.36	55.58	54.31	198.46	19	19	48
Glutamate dehydrogenase	71	KMZ75350.1	MT	UR	7.06	6.76	43.16	44.36	166.51	13	3	34
Glutamate-ammonia ligase	73	KMZ72531.1	CY	UR	5.34	5.42	42.94	39.39	246.87	27	7	68
Glycine dehydrogenase (D)	8	KMZ58990.1	MT	NA	6.48	6.15	99.04	113.46	217.34	33	27	40
Glycine hydroxymethyltransferase	53	KMZ69888.1	MT	UR	7.09	8.79	54.45	57.82	217.49	24	24	55
Glycine hydroxymethyltransferase	21	KMZ63006.1	MT	NA	8.14	7.22	53.61	51.84	196.68	24	24	59
Glyoxylate reductase	57	KMZ70332.1	PX	UR	7.47	6.56	44.88	41.68	205.97	19	3	50
Inositol-3-phosphate synthase	31	KMZ57169.1	CY	NA	5.19	5.84	64.24	64.95	232.12	22	9	45
M1P guanylyltransferase	51	KMZ56564.1	CY	UR	6.76	7.12	39.42	39.71	133.93	7	7	22
Malate dehydrogenase	69	KMZ65231.1	CL	UR	7.47	6.32	38.52	35.56	224.96	27	27	62
Malate dehydrogenase	52	KMZ65231.1	CL	UR	7.08	6.53	38.89	35.63	250.27	31	8	64
Methionine synthase	11	KMZ76082.1	CL	NA	6.2	5.92	84.29	84.67	305.86	34	10	53
NAD-epimerase/dehydratase	66	KMZ71456.1	MT	UR	8.74	8.93	38.43	42.80	151.94	9	6	53
NADP-GA3P dehydrogenase	3	KMZ74191.1	CL/CY	NA	6.76	6.76	55.24	53.16	175.96	15	15	34
Nucleoside-diphosphate kinase	82	KMZ65765.1	MT	DR	5.22	5.91	14.66	16.50	145.36	6	2	41
Phosphoglucosamine mutase	29	KMZ74697.1	CL	NA	5.52	5.10	65.54	63.11	231.63	25	8	57
Phosphoglycerate kinase	44	KMZ64101.1	CL	UR	5.74	8.30	45.68	50.33	216.51	31	23	61
Pyrroline-5-carboxylate reductase	6	KMZ57016.1	CY	NA	7.44	6.91	26.21	28.88	191.05	10	10	46
Transketolase	67	KMZ75731.1	CL	UR	5.74	5.93	77.89	81.03	281.81	43	17	69
Triose-phosphate isomerase	39	KMZ74865.1	CL/CY	UR	5.35	5.12	26.19	27.34	183.38	12	3	55
UDP–glucose pyrophosphorylase	32	KMZ76338.1	CY	NA	5.56	5.20	58.39	51.68	248.96	21	21	58
V-ATPase subunit E	62	KMZ70197.1	CL	UR	7.64	7.22	29.26	26.70	153.29	14	14	49
**PHOTOSYNTHESIS**												
Ferredoxin-NADP reductase	50	KMZ70342.1	CL	UR	6.35	8.68	35.93	40.57	214.76	38	28	62
LHCI Chl a/b binding protein3	85	KMZ65488.1	CL	DR	6.13	8.96	22.62	29.72	142.97	9	6	36
OEE protein	83	KMZ65829.1	CL	DR	5.83	8.65	23.09	28.02	204.83	18	14	53
OEE protein 3	90	KMZ70530.1	CL	DR	8.93	9.58	14.83	24.58	175.92	9	6	41
PSI reaction center subunit N	86	KMZ63587.1	CL	DR	6.94	9.35	10.80	18.44	147.34	8	1	34
RuBisCO activase	42	KMZ57183.1	CL	UR	5.71	6.13	42.57	49.24	235.12	22	11	59
RuBisCO large subunit, P	77	AIZ98377.1^†^	CL	UR	6.35	6.09	52.63	50.21	175.83	9	1	19
RuBisCO large subunit, P	68	AIZ98377.1^†^	CL	UR	6.11	6.09	52.86	50.21	167.87	13	1	26
RuBisCO large subunit, P	49	AIZ98377.1^†^	CL	UR	6.03	6.09	52.43	50.21	170.91	14	1	29
RuBisCO large subunit, P	4	AIZ98377.1^†^	CL	NA	6.42	6.09	116.12	50.21	265.64	22	1	33
RuBisCO small subunit	72	KMZ72699.1	CL	UR	6.11	8.24	12.91	20.41	187.25	21	1	65
RuBisCO small subunit	48	KMZ72699.1	CL	UR	6.02	8.24	12.90	20.41	152.76	13	1	86
RuBisCO small subunit	43	KMZ72699.1	CL	UR	6.39	8.24	12.94	20.41	166.26	19	11	65
**ANTIOXIDANT AND DEFENSE SYSTEM**												
Aldehyde dehydrogenase	28	KMZ61155.1	MT	NA	6.41	6.4	58.84	58.05	153.35	9	8	18
Ascorbate peroxidase	40	ALK24273.1	PX	UR	5.56	5.45	26.57	27.72	231.60	17	8	65
Ascorbate peroxidase	14	AII01419.1	PX	NA	6.46	5.66	26.5	27.39	164.45	10	3	46
Ascorbate peroxidase	16	ALK24273.1	PX	NA	5.45	5.45	26.71	27.72	218.15	10	6	58
Ascorbate peroxidase 4	61	KMZ62361.1	CL	UR	7.68	8.87	26.23	35.11	214.65	24	19	57
Ascorbate peroxidase 4	20	KMZ62361.1	CL	NA	8.97	8.87	26.36	35.11	232.43	24	19	58
Catalase Peroxidase	54	KMZ68871.1	PX	UR	7.16	6.29	54.63	56.8	248.42	36	9	56
Cysteine synthase	34	KMZ71691.1	CL/MT	NA	5.63	5.36	38.61	35.18	161.36	8	3	29
GDP-mannose 3,5-epimerase 1	33	KMZ62116.1	GB	NA	5.89	5.92	48.38	42.72	246.15	25	8	64
Germin-like protein 2-1	89	KMZ73730.1	CW	DR	7.28	6.41	21.05	23.34	110.07	7	7	30
Glutathione Peroxidase	35	KMZ63257.1	CL/MT	NA	6.46	6.59	18.2	18.3	131.21	7	7	41
GSH-S-transferase F7	24	KMZ61632.1	CY	NA	6.71	5.44	22.9	24.38	198.73	21	20	56
GSH-S-transferase F7, Phi class	81	KMZ61632.1	CY	DR	5.65	5.44	23.78	24.38	144.73	8	8	42
GSH-S-transferase F9	23	KMZ60880.1	CY	NA	6.13	5.46	23.52	23.93	230.12	18	17	84
Lactoylglutathione lyase	79	KMZ64007.1	CY	UR	5.49	5.37	32.57	32.75	202.91	12	10	49
Lipoxygenase (13-LOX)	27	KMZ68413.1	CY	NA	5.47	5.61	98.07	104.76	183.25	15	6	22
MDHA reductase	76	KMZ72399.1	CY	UR	5.64	5.24	48.3	46.64	229.94	22	7	54
MDHA reductase	5	KMZ72399.1	CY	NA	5.78	5.24	48.38	46.64	275.18	30	11	68
Peroxidase	65	KMZ56929.1	CY	UR	8.66	8.29	38.73	36.33	127.06	7	7	26
Peroxidase	63	KMZ56929.1	CY	UR	8.07	8.29	35.34	36.33	175.14	10	10	34
Peroxidase	19	KMZ69590.1	CY	NA	8.74	8.69	33.32	34.55	109.57	5	5	22
Superoxide dismutase [Cu-Zn]	**92**	KMZ60238.1	CL	DR	6.03	5.76	15.3	15.51	147.91	4	2	33
**GENETIC INFORMATION AND PROCESSING**												
ATP-Zn metalloprotease FtsH 4	78	KMZ70870.1	CL	UR	5.37	5.77	75.16	74.17	275.62	32	8	49
Chaperone protein ClpB 1	26	KMZ64529.1	CL	NA	5.66	5.93	91.69	102.11	235.95	56	2	65
Chaperonin 60 subunit beta 2,	30	KMZ69424.1	CL	NA	5.25	5.59	64.24	64.70	249.79	30	5	53
Chaperonin-20 kDa	80	KMZ69941.1	CL	DR	5.74	8.49	24.95	27.09	203.38	12	10	46
Elongation factor Tu	46	KMZ72737.1	CL	UR	5.81	6.25	46.17	51.46	270.07	35	7	70
Heat shock protein 70	1	KMZ71868.1	CL	NA	5.3	5.47	75.5	68.79	231.46	24	1	39
Heat shock protei-STI1	10	KMZ64384.1	NU	NA	6.61	5.79	75.97	65.95	206.59	32	29	57
Heat shock protei-STI1	12	KMZ64384.1	NU	NA	6.49	5.79	76.26	65.95	298.29	29	27	53
NAC subunit beta	93	KMZ60575.1	NU	DR	7.17	7.92	18.61	16.35	106.41	6	6	51
Polyubiquitin 11	88	KMZ73934.1	NU	DR	7.2	6.75	9.41	17.27	141.48	6	1	31
PPIase-cyclophilin superfamily	91	KMZ56118.1	CY	DR	8.1	8.37	15.75	17.83	40.06	1	1	8
**SECONDARY METABOLISM**												
Dihydroflavonol-4-reductase	75	KMZ70095.1	CL/GB	UR	5.71	5.5	39.46	36.9	199.78	17	3	61
Dihydroflavonol-4-reductase	41	KMZ70095.1	CL/GB	UR	5.57	5.5	39.81	36.9	144.92	10	2	26
Isoflavone reductase	45	KMZ72723.1	CY	UR	5.73	5.13	37.58	35.23	279.64	30	17	81
Putative Cinnamoyl-CoA reductase	15	KMZ62526.1	GB	NA	5.89	5.77	30.54	35.76	118.23	4	4	17
**SIGNALING AND VESICLE TRAFFICKING**												
ARF family protein	87	KMZ67128.1	CY/ER	DR	7.1	6.43	20.93	22.45	101.14	4	4	25
α-SNAP	36	KMZ58533.1	ER/GB	UR	5.01	4.97	34.08	32.87	132.39	5	1	21
**OTHERS**												
Actin-97	7	KMZ67762.1	CY	NA	5.2	5.31	38.62	41.7	135.99	4	4	14
Annexin	18	KMZ64931.1	CY	NA	8.42	8.24	33.08	35.85	147.34	13	13	39
Hypothetical protein	84	KMZ58302.1	PM	DR	6.05	6.10	27.42	21.44	139.27	7	7	45

a *Subcellular location of proteins was predicted using the online Plant-mPLoc server (http://www.csbio.sjtu.edu.cn/bioinf/plant-multi/)*;

b *exclusive unique peptide count*;

c *exclusive unique spectrum count*;

* all accession matches to Zostera marina except

# (match with Z. noltei) and

† *(match with Z. angustifolia); obs, observed; theo, theoretical*.

*pI, isoelectric point; Mr, molecular weight; UR, up-regulated; DR, down-regulated; NA, newly appeared; PX, peroxisome; CL, chloroplast; CY, cytoplasm, MT, mitochondria; GB, golgi body; NU, nucleus; CW, cell wall; ER, endoplasmic reticulum; PM, plasma membrane; SC, sequence coverage; P, partial; (P), phosphorylating; (D) decarboxylating; M1P, Mannose-1-phosphate; GA3P, Glyceraldehyde-3-phosphate; NAD, nicotinamide adenine dinucleotide; UDP, uridine diphosphate; OEE, oxygen evolving enhancer; PS, photosynthesis system; LHC, light harvesting complex; MDHR, monodehydroascorbate reductase; GSH, glutathione; NAC, nascent polypeptide-associated complex, SNAP, alpha-soluble NSF attachment protein; PPIase, peptidyl-prolyl cis/trans isomerase*.

Within the ECBM group, 23 of 38 protein spots (60%) were up-regulated with a fold change ranged from 2.2 to 4.06 (Figure 2A, Supplementary Figure 2). The photo-respiratory protein, glyoxylate/hydroxypyruvate reductase B (Gx/HPR, spot 57), was the most significantly up-regulated protein in SSL conditions compared to the Control (*F* = 47.05, *p* = 0.001, Cohen's *d* = 3.54.). Other up-regulated proteins in the ECBM category were associated with the Calvin Benson cycle (C_3_ cycle) and/or glycolysis (EMP), such as glyceraldehyde-3-phosphate dehydrogenase (GA3PDH, spot 56, 59, 60, and 64), phosphoglycerate kinase (PGAK, spot 44), transketolase (TK, spot 67), triose phosphate isomerise (TPI, spot 39), fructose-bisphosphate aldolase (FBA, spot 13 and 74), and malate dehydrogenase (MDH, spot 52, and 69) (Table 1, Figure 2B, Supplementary Figure 2). The appearance of the same GA3PDH and MDH proteins at different observed p*I*s in the gel suggested the possible occurrence of post translational modifications. Proteins linked with energy production such as H (+)-transporting two-sector ATPase (F-APTase, spot 38), ATP synthase beta subunit (ATPase-β, spot 37), V-type proton ATPase subunit E (V-ATPase E, spot 62) were also up-regulated by 2.4–2.9 fold as compared to the Control (Table 1, Figure 2B, Supplementary Figure 2). Proteins involved in amino acid metabolism such as aspartate transaminase (AT, spot 58), adenosylhomocysteinase (AHCY, spot 47), glutamate decarboxylase (GDC, spot 70), glutamate-ammonia ligase (also known as glutamate synthase; GAL/GS, spot 73), glutamate dehydrogenase (GDH, spot 71) and glycine hydroxymethyltransferase (GlyHMT, spot 53) were UR proteins. Two mitochondrial localized proteins namely dihydrolipoyl dehydrohenase (mitochondrial) (mtLPD, spot 55) and NAD-epimerase/dehydratase (spot 66) together with a cell wall protein named manose-1-phosphate guanyltransferase (MPGT, spot 51) were also up-regulated significantly.

Among NA, proteins belonging to TCA and the EMP pathway such as NADP dependent- glyceraldehyde-3-phosphate dehydrogenase (NADP-GA3PDH, spot 3), citrate (Si)-synthase/succinate—CoA ligase (CS, spot 9), aconitate hydratase (AH, spot 25) were included. Other NA proteins likely to be involved in the glycine cleavage complex included Gly/HMT (spot 21), glycine dehydrogenase decarboxylating (GlyDH, spot 8), and aminomethyltransferase (AMT, spot 17). A few more proteins involved in amino acid metabolism also appeared including: pyrroline-5-carboxylate reductase (P5CR, spot 6) and methionine synthase (MS, spot 11) (Table 1).

Within the photosynthesis process (PS) group, 8 of the 13 proteins (61%) were up-regulated, one newly appeared and four were down-regulated during conditions of SSL compared to the Control (Figure 2A). The UR proteins included the ribulose-1,5-bisphosphate carboxylase/oxygenase large subunit, partial (RuBisCO-L; spot 4, 49, 68, and 77), the RuBisCO small subunit (RuBisCO-S, spot 48, and 72), RuBisCO activase (spot 42), ferrodoxin-NADP reductase (FNR, spot 50) and the thylakoid lumen 29 kDa protein (APX 4, spot 61) (Figure 2B). All RuBisCO large subunits (spot 49, 68, and 77) were enhanced remarkably by 5- to 6-fold (*F* = 33.62, 19.41, 46.26 respectively, *p* < 0.001, Cohen's d = 9.4, 7.7, and 6.3 respectively) (Table 1, Figure 2B, Supplementary Figure 2). The NR protein- RuBisCO large subunit (spot 4), with a different p*I* on the gel, suggested that there may exist a proteoform of the same protein. Down-regulated proteins included: the oxygen evolving enhancer protein (OEE, spot 83), OEE -3 (spot 90); photosystem I reaction center subunit N (PS1-N, spot 86), and light-harvesting complex I chlorophyll a/b binding protein 3 (LHC1-CAB3, spot 85; Table 1, Figure 2C, Supplementary Figure 2).

In the antioxidant and defense function (ADS) group, 7 of 22 proteins (32%) that were up-regulated (1.7- to 4-fold) included: cytoplasmic monodehydroascorbate reductase (MDHAR, spot 76, 4-fold increase, *F* = 38.74, *p* > 0.002, Cohen's *d* = 6.25), peroxidase (POXs, spot 63, and 65), and lactoglutathione lyase (LGL, spot 79). Other UR proteins localized to peroxisomes were catalase peroxidase (CAT, spot 54) and ascorbate peroxidase (APX, spot 40). Apart from the UR proteins, 12 of 22 proteins (55%) were NA, of which four proteins were found to be involved in glutathione metabolism (see spots 23, 24, 34, and 35) (Table 1, Figure 2B, Supplementary Figure 2). Other proteins involved in reactive oxygen species (ROS) mediated signaling were also identified: lipoxygenase (LOX, spot 27), ROS detoxification such as aldehyde dehydrogenase (ALDH, spot 28), peroxisomal APXs (spot 14, 16), cytoplasmic POX (spot 19), and plastid APX4 (spot 61) (Table 1, Figure 2B). Three remaining proteins (14%) were identified as superoxide dismutase [Cu-Zn] (SOD-Cu/Zn, spot 92), germin-like protein 2-1 (GLP, spot 89) and glutathione S-transferase F7, Phi class (GST F7, spot 81) and were significantly down-regulated (Table 1, Figure 2C, Supplementary Figure 2).

In the group of genetic information processing (GIP), most of the differentially regulated proteins belonged to protein folding, sorting and degradation functions. In this category, 5 of 11 proteins (45%) were NA, and two (18%) were up-regulated (Figure 2A). The NA proteins included heat shock protein 70 kDa (HSP70, spot 1), heat shock protein STI1 (spot 10 and 12), chaperone protein ClpB 1 (chap- ClpB1, spot 26) and chaperone 60 subunit β2 (chap60 β2, spot 30) (Figure 2B, Table 1). The ATP-dependent zinc metalloprotease FtsH 4 (FtsH4, spot 78) and elongation factor Tu (EF-Tu, spot 46) were two of the most up-regulated proteins with a >2.6-fold change during SSL conditions (Figure 2B, Supplementary Figure 2, Table 1). Four of 11 proteins (36%) were found down-regulated. These included polyubiquitin 11 (spot 88), nascent polypeptide-associated complex subunit beta (NACβ, spot-93), among others (Table 1, Supplementary Figure 2).

Four proteins were classified as linked to secondary metabolism, including dihydroflaonol-4-reductase (DHFR, spot 75, and 41) and isoflavone reductase (IFR, spot 45), that are involved in anthocyanin pigment and flavonoids synthesis. Their expressions were significantly increased to ≥4-fold under SLL conditions compared to the Control (Figure 2B, Supplementary Figure 2, Table 1). The putative Cinnamoyl-CoA reductase involved in lignin synthesis (CCR, spot15) was the only NA protein identified in this category. In the group of signaling and vesicular trafficking (SVT), a protein identified as the alpha-soluble NSF attachment protein (αSNAP, spot) was up-regulated. However, the ADP-ribosylation factor family protein (ARFP, spot 87) was observed as down-regulated (Figure 2C, Table 1) under SSL conditions.

#### Comparative proteome analysis of plants exposed to control and LL conditions

Among the 40 protein spots that were differentially regulated under LL conditions, compared to control light conditions, a total of 36 spots, with defined accession IDs, were successfully retrieved using Batch Entrez NCBI database after filtering duplicate IDs (4).

Of the 40 differentially regulated protein spots, 32 were DR (80%) under LL conditions 5 were UR (12.5%) and 3 were NA (7.5%) (Table 2, Figure 3A). The most sensitive biochemical pathways in the *Z*. *muelleri* in response to LL conditions were ECBM and PS that represent 38 and 28%, respectively, of total differential regulated proteins (Figure 3A). Other functional categories of differential regulated proteins were represented as GIP (10%), SVT (8%), ADS (8%), SM (3%), and others (8%).

**Table 2:** **Identification of proteins with varied normalized volumes between saturated light (SL, Control; 200 μmol photons m^−2^ s^−1^) and light limitation (LL, 20 μmol photons m^−2^ s^−1^) stress of *Zostera muelleri***.

**Protein**	**Spot no**.	Accession^*^	**Subcellular localization^a^**	**Regulation**	**pI**		**Mr (kD)**		**Score (PEAKS)**	**Pep^b^**	**Uni ^c^**	**SC**
					**Obs**.	**Theo**.	**Obs**.	**Theo**.				
**ENERGY, CARBOHYDRATE, AND BIOMOLECULE METABOLISM**												
Adenine nucleotide α hydrolases	17	gb|KMZ58089.1	CL/NU	DR	6.67	5.63	17.25	18.38	77.3	3	3	22
Adenylate kinase	36	gb|KMZ75520.1	CL	DR	8.59	6.31	27.38	26.30	153.75	12	4	54
Aspartate transaminase	30	gb|KMZ73817.1	CL	DR	7.91	7.72	40.96	44.36	235.43	39	6	58
ATP synthase beta subunit, P	4	gb|AAK72873.1 ^#^	CL	UR	5.12	5.17	55.2	51.26	255.78	34	6	62
Ferredoxin-NADP reductase	39	gb|KMZ70342.1	CL	DR	6.45	8.68	35.93	40.57	214.76	38	28	62
GA3P dehyrogenase (P)	35	gb|KMZ61796.1	CL	DR	8.4	7.63	38.3	43.31	262.32	29	10	60
GA3P dehyrogenase (P)	33	gb|KMZ64911.1	CL	DR	7.76	6.97	38.22	36.47	180.63	17	1	45
GA3P dehyrogenase (P)	34	gb|KMZ61796.1	CL	DR	7.61	7.63	37.85	43.31	264.39	36	12	64
Gamma carbonic anhydrase 1	1	gb|KMZ56823.1	MT	NR	5.71	5.76	28.18	26.0	140.9	4	2	23
Glutamate-ammonia ligase	19	gb|KMZ72531.1	MT	DR	5.34	5.42	42.94	39.39	246.87	27	7	68
Glycine hydroxymethyltransferase	29	gb|KMZ69888.1	MT	DR	7.37	8.79	54.57	57.82	263.12	48	48	70
Malate dehydrogenase	26	gb|KMZ62786.1	CL	DR	6.92	6.32	36.49	35.56	178.87	16	4	58
NAD epimerase/dehydratase	37	gb|KMZ71456.1	MT	DR	8.74	8.93	38.43	42.80	151.94	9	6	53
Nucleoside-diphosphate kinase	11	gb|KMZ65765.1	MT	DR	6.32	5.91	15.2	16.50	99.0	2	1	20
Phosphoglycerate kinase	18	gb|KMZ64101.1	CL	DR	5.74	8.3	45.68	50.33	257.28	42	23	68
Triose-phosphate isomerase	25	gb|KMZ74865.1	CL/CY	DR	5.35	5.12	26.19	27.34	183.38	12	3	55
**PHOTOSYNTHESIS**												
LHCII Chl a/b binding protein	3	gb|KMZ57168.1	CL	NR	5.28	6.75	33.72	27.64	97.43	2	2	12
OEE protein 3	2	gb|KMZ70530.1	CL	NR	7.52	9.58	15.76	24.58	175.92	9	6	41
OEE-PsbP	40	gb|KMZ57551.1	CL	DR	7.4	8.76	23.02	27.68	200.2	13	11	36
PsbP-like protein 1	21	gb|KMZ62962.1	CL	DR	6.67	9.22	17.25	28.18	193.83	14	8	51
RuBisCO	27	gb|KMZ56152.1	CL	DR	5.42	8.55	13.26	20.50	153.36	12	1	59
RuBisCo activase	28	gb|KMZ57183.1	CL	DR	5.71	6.13	42.57	49.24	235.12	22	11	59
RuBisCO large subunit, P	38	gb|AIZ98377.1^†^	CL	DR	6.11	6.09	52.43	50.21	170.91	14	1	29
RuBisCO small subunit	31	gb|KMZ72699.1	CL	DR	5.73	8.24	12.94	20.41	160.02	16	1	64
RuBisCO small subunit	20	gb|KMZ72699.1	CL	DR	6.11	8.24	12.91	20.41	187.25	21	1	65
RuBisCO small subunit	32	gb|KMZ72699.1	CL	DR	6.02	8.24	12.91	20.41	153.36	14	1	86
**ANTIOXIDANT AND DEFENSE SYSTEM**												
Ascorbate peroxidase 4	22	gb|KMZ62361.1	CL	DR	7.68	8.87	26.23	35.11	214.65	24	19	57
Peroxidase	23	gb|KMZ56929.1	CY	DR	8.07	8.29	35.34	36.33	175.14	10	10	33
Peroxidase	24	gb|KMZ56929.1	CY	DR	8.66	8.29	38.73	36.33	127.06	7	7	27
**GENETIC INFORMATION PROCESSING**												
eIF-5A-2 protein	16	gb|KMZ65349.1	CL	DR	5.45	5.59	17.56	17.30	197.7	9	6	65
NAC subunit beta	14	gb|KMZ60575.1	NU	DR	7.17	7.92	18.61	16.35	106.41	6	6	51
PPIase-cyclophilin superfamily	5	gb|KMZ56118.1	CY	UR	8.1	8.37	15.75	17.83	40.06	1	1	8
pTAC16 protein	15	gb|KMZ73091.1	CL	DR	7.69	8.98	55.54	53.98	179.42	14	2	24
**SECONDARY METABOLISM**												
Isoflavone reductase	13	gb|KMZ72723.1	CY	DR	5.73	5.13	37.58	35.23	279.64	30	17	81
**SIGNALING AND VESICLE TRAFFICKING**												
ARF	12	gb|KMZ70858.1	MT	DR	6.9	6.43	18.59	20.64	104.81	6	6	31
ARF family protein	7	gb|KMZ67128.1	CY/ER	UR	7.1	6.43	20.93	22.45	101.14	4	4	25
α-SNAP	6	gb|KMZ58533.1	ER/GB	UR	5.01	4.97	34.08	32.87	132.39	5	1	21
**OTHERS**												
Carnitine operon protein CaiE	10	gb|KMZ68943.1	MT	DR	7.82	9.14	23.34	28.97	188.38	12	6	59
Hypothetical protein	8	gb|KMZ58302.1	PM	UR	6.05	6.10	27.42	21.44	139.27	7	7	45
Putative Actin	9	gb|KMZ55988.1	CY	DR	5.51	5.31	47.87	41.72	162.1	13	2	46

a *Subcellular location of proteins was predicted using the online Plant-mPLoc server (http://www.csbio.sjtu.edu.cn/bioinf/plant-multi/)*;

b *exclusive unique peptide count*;

c *exclusive unique spectrum count*;

* all accession matches to Zostera marina except

# (match with Z. noltei) and

† *(match with Z. angustifolia); obs, observed; theo, theoretical*.

*pI, isoelectric point; Mr, molecular weight; UR, up-regulated; DR, down-regulated; NA, newly appeared; PX, peroxisome; CL, chloroplast; CY, cytoplasm, MT, mitochondria; GB, golgi body; NU, nucleus; CW, cell wall; ER, endoplasmic reticulum; PM, plasma membrane; SC, sequence coverage; P, partial; (P), phosphorylating; GA3P, glyceraldehyde-3-phosphate; NAD, nicotinamide adenine dinucleotide; OEE, oxygen evolving enhancer; PS, photosynthesis system; LHC, light harvesting complex; NAC nascent polypeptide-associated complex, SNAP, alpha-soluble NSF attachment protein; ARF, ADP-ribosylation factor; pTAC, protein plastid transcriptionally active; eIF, eukaryotic elongation factor*.

Within the ECBM group, 13 of 15 (86%) differentially regulated proteins were DR, with one UR, and one NA (7%). Among the DR proteins, identified proteins were mostly associated with C3 cycle and amino acid metabolism. These proteins include TPI (spot 25), PGK (spot 18), and GA3PDH (spot 33–35) and MD (spot 26) (Table 2, Figure 3C, Supplementary Figure 3). Proteins involved in amino acid metabolism included GAL/GS (spot 19), GyHMT (spot 29), AT (spot 30) (Table 2, Figure 3C, Supplementary Figure 3). Other proteins involve in maintaining nucleotide pool in cell were also found to be significantly down-regulated (Table 2, Figure 3C, Supplementary Figure 3). The only UR protein within this category was ATPaseβ (spot 4, 2.5 fold, *F* = 34.54, *p* < 0.001, Cohen's *d* = 7.32), while gamma carbonic anhydrase 1 protein (γ-CA1, spot 1) was the only NA protein (Figure 3B).

PS was the second most altered group under LL conditions. Nine of 11 (82%) proteins were DR and only two proteins (18%) were NA. Among the DR proteins, RuBisCO (spot 27), RuBisCO activase (spot, 28), RuBisCO-S (spot 20, 31, and 32), RuBisCO-L, partial (spot 38), FNR (spot 39), OEE-PsbP (spot 40), and PsbP-like protein 1 (PPL1, spot 21) were major photosynthetic proteins. OEE protein 3 (spot 2) and the LHCII–CAB protein (spot 3) were two new up-regulated proteins in this category.

Several proteins belonging to other functional categories were down-regulated (Table 2, Figures 3B,C, Supplementary Figure 3). However, proteins such as PPIase (spot 5, 2.9 fold, *F* = 25.50, *p* < 0.001), α-SNAP (spot 6, 4 fold, *F* = 19.27, *p* < 0.002), and the ARF family protein (spot 7, 1.7 fold, *F* = 21.24, *p* < 0.01) were up-regulated during LL conditions when compared to the Control.

## Discussion

**Figure 4:**
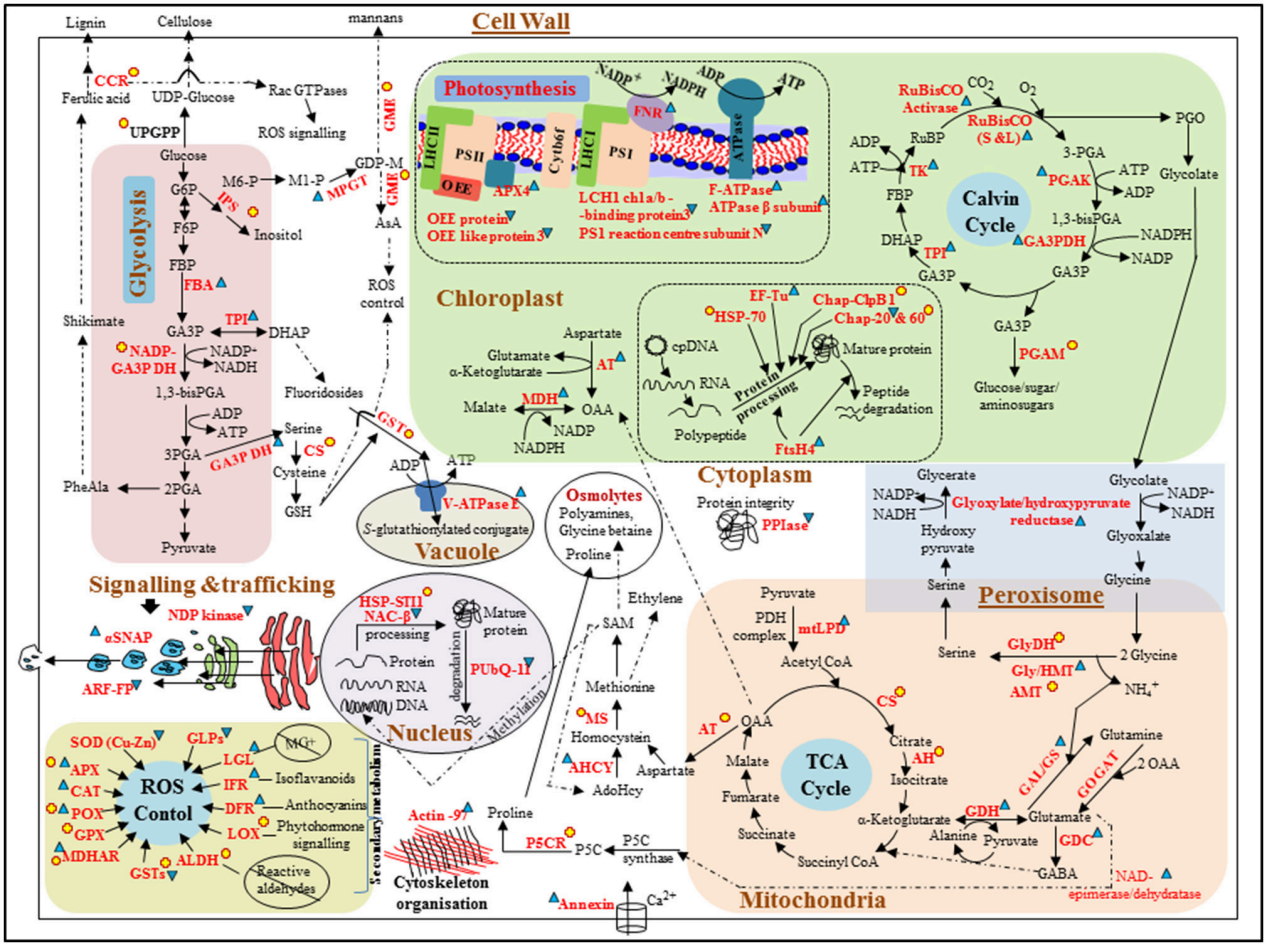
**Schematic representation of differentially expressed proteins involved in different metabolic pathways/cellular processes in *Zostera muelleri* to tolerate super-saturating light (SSL, 600 μmol photons m^−2^s^−1^) irradiance condition**. Differentially expressed proteins are represented in red. Proteins that were up-regulated and down-regulated are followed by 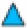 and 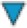 respectively. Newly appeared proteins are followed by 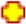. Dotted line arrows represent the indirect connection of protein/enzyme/metabolite to diverse metabolic processes. *Protein abbreviations are as followed*: OEE: oxygen evolving enhancer protein; LHCI/II-CAB3: light harvesting complex of photosystem I/II-chlorophyll a/b binding proteins; F-ATPase: H(+)-transporting two-sector ATPase; ATPaseβ subunit: ATP synthase β subunit, APX4: ascorbate peroxidase 4; FNR: ferredoxin-NADP reductase; RuBisCO (S and L): ribulose-1,5-bisphosphate carboxylase/oxygenase large and small subunits; PGAK: phosphoglycerate kinase; GA3PDH: glyceraldehyde-3-phosphate dehydrogenase; TPI: triose-phosphate isomerase; TK: transketolase; PGAM: phosphoglucosamine mutase; AT: aspartate transaminase; MDH: malate dehydrogenase; HSP70: heat shock protein 70; EF-Tu: elongation factor Tu; Chap-ClpB1: chaperone protein ClpB 1; Chap 20 and 60: chaperone protein 20 and 60; FtsH4: ATP-dependent zinc metalloprotease FtsH 4; CCR: cinnamoyl-CoA reductase; UPGPP: UTP–glucose-1-phosphate uridylyltransferase; IPS: inositol-3-phosphate synthase; MPGT: mannose-1-phosphate guanylyltransferase 1; GME: GDP-mannose 3,5-epimerase 1; FBA: fructose-bisphosphate aldolase; CS: cysteine synthase; V-ATPaseE: V-type proton ATPase subunit E; PPIse: peptidyl-prolyl cis-trans isomerase-cyclophilin superfamily; GlyDH: glycine dehydrogenase; Gly/HMT: glycine/hydroxymethyltransferase; AMT: aminomethyltransferase; GAL/GS: glutamate-ammonia ligase/glutamate synthase; GOGAT: glutamine oxoglutarate aminotransferase; GDC: glutamate decarboxylase; GDH: glutamate dehydrogenase; mtLPD: dihydrolipoyl dehydrogenase; CS: cysteine synthase; AH: aconitate hydratase; P5CR: pyrroline-5-carboxylate reductase; AHCY: adenosylhomocysteinase; MS: methionine synthase; PUbQ-11: polyubiquitin 11; HSP-STI1: heat shock protein STI1; NACβ: nascent polypeptide-associated complex subunit beta; SOD(Cu-Zn): Superoxide dismutase (Cu-Zn); APX: ascorbate peroxidase; CAT: catalase; POX: peroxidase; GPX: glutathione peroxidase; MDHAR: monodehydroascorbate reductase; ALDH: aldehyde dehydrogenase; LOX: lipoxygenase; DFR: dihydroflavonol-4-reductase; IFR: isoflavone reductase-like protein; LGL: lactoylglutathione lyase; GLPs: putative germin-like protein 2-1; NDP kinase: nucleoside-diphosphate kinase; SNAP: alpha-soluble NSF attachment protein; ARF: ADP-ribosylation factor (family) protein. *Metabolite abbreviations are as followed*: G6P: glucose-6-phosphate; F6P: fructose-6-phosphate; FBP: fructose 1, 6 bis-phosphate; GA3P: glyceraldehyde-3-phosphate; DHAP: dihydroxyacetone phosphate; PGA: phosphoglyceraldehyde; GDPM: GDP mannose; AsA: ascorbate; GSH: glutathione; OAA: oxaloacetate; PGO: phosphoglycolate; GABA: gamma aminobutyric Acid.

**Figure 5:**
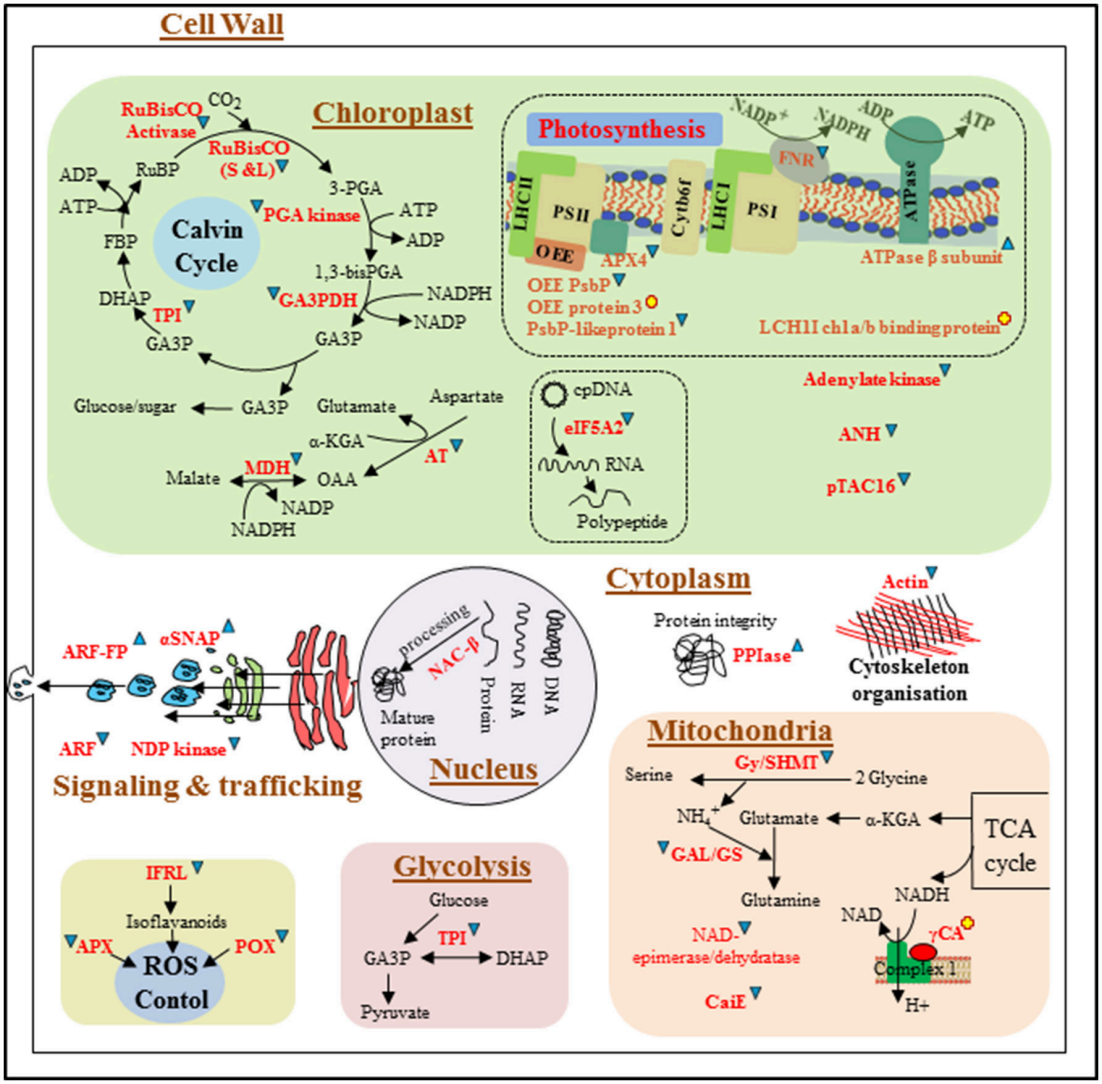
**Schematic representation of differentially expressed proteins involved in different metabolic pathways/cellular processes in *Z. muelleri* in response to limited light (LL, 20 μmol photons m^−2^s^−1^) irradiance condition**. Differentially expressed proteins are represented in red. Proteins that were up-regulated and down-regulated are followed by 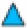 and 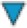 respectively. Newly appeared proteins are followed by 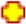. Dotted line arrows represent the indirect connection of protein/enzyme/metabolite to diverse metabolic processes. Protein abbreviations are given in Figure 4, however few protein abbreviations which are not represented in Figure 4 are as followed: ANH: adenine nucleotide alpha hydrolases-like superfamily; pTAC16: protein plastid transcriptionally active 16; eIF-5A: eukaryotic translation initiation factor 5A-2; γCA: gamma carbonic anhydrase; CaiE: carnitine operon protein CaiE. Metabolite abbreviations are as followed in Figure 4.

A proteomic approach was used to identify key protein markers of vulnerability to environmental stress conditions (e.g., extreme light) in the marine seagrass *Z. muelleri*. It was found that most differentially regulated proteins were up-regulated or changed from zero to positive expression (newly appeared proteins), compared to the Control. These proteins were related to diverse metabolic pathways (mainly carbon, amino acid, C3, glycolysis, TCA, photorespiration, antioxidant defense system, and secondary metabolism; Figure 4). In contrast, LL conditions mostly induced the down-regulation of proteins, compared to Control conditions (Figure 5). The changes in the *Z. muelleri* proteome are discussed below according to known light stress tolerance/acclimation mechanisms. The discussion is based principally on current knowledge of higher plants, and on the results coming from the rare proteomic approaches on seagrasses.

### Limiting photosynthetic damage while ensuring an adequate supply of energy and reducing equivalents for the calvin cycle

Photosynthesis is the most sensitive cellular process that is affected by light stress. *Z. muelleri* grown under SSL conditions had a significantly higher rETR_max_ value, as compared to *Z. muelleri* grown under LL and control conditions, indicating a higher photosynthetic efficiency. The changes in Ek and rETR_max_ in SSL conditions suggested that the plants acclimated their light-harvesting apparatus and photosystem arrangements to cope with such irradiance shifts, which is in agreement with a previous study (Dattolo et al., 2014). Remarkably, only SSL conditions induced a significant up-regulation of proteins involved in photosynthetic processes, such as FNR, RuBisCO (both S and L subunits), RuBisCO activase, and APX4 (a thylakoid lumen 29 kDa protein, TL29). Instead, other structural proteins of the photosystems I and II (PSI and PSII), such as the oxygen evolving enhancer proteins (OEEs), PSI-N, and LHCI-CAB were down-regulated. Three classes of OEEs have been described: OEE1 (PsbO), OEE2 (PsbP), and OEE3 (PsbQ). These proteins are peripherally bound to the PSII on the lumenal side of the thylakoid membrane, and play important roles in light-induced water oxidation, therefore maintaining PSII integrity. However, the subunits of the PSII complex can be easily dissociated under stress (Järvi et al., 2013). PSI-N is the only extrinsic PSI subunit on the lumenal side of the thylakoid membrane, and it was shown to be important for efficient electron transfer from plastocyanin to P700 (Järvi et al., 2013). In the present study, down-regulation of the OEE like protein, OEE3 and PSI-N, not only suggested partial damage to the photosystem, but also impairment in the linear electron flow from PSII to PSI, possibly due to the generation of ROS during plant exposure to SSL conditions. Despite the down-regulation of OEEs and LHC1-CAB3, SSL-stressed *Z. muelleri* displayed high rETR_max_, Ek together with up-regulation of chloroplastic FtsH4, suggesting that the PSII is being repaired. FtsH4 is a Zn^2+^-dependent metalloprotease that plays a critical role in PSII repair; it degrades photodamaged D1 reaction center proteins, unassembled cytochrome *b*_6_*f* Rieske FeS proteins, and LHCII proteins (Järvi et al., 2013). FtsH proteases degrade other unassembled proteins under stress conditions, and prevent charge separation and singlet oxygen production, thus protecting the PSII core structures from light stress damage (Yoshioka and Yamamoto, 2011). Further, up-regulation of APX4 during SSL conditions also supports the assembly and repair of PSII, due to its involvement in stabilizing and/or assembling the lumenal side of PSII during stress conditions (Granlund et al., 2009). LHCI-CAB proteins facilitate light absorption and transfer of the excitation energy to the reaction centers of PSI, for the reduction of NADP^+^ to NADPH. Down-regulation of the LHCI-CAB3 protein during SSL conditions could be an acclimation strategy to avoid excess light absorption and prevent PSI photodamage. Shrinking of the PS antenna, a strategy used to prevent photodamage due to excessive light, has been reported in higher plants and in the seagrass *Posidonia oceanica* (Dattolo et al., 2013).

Conversely, the appearance of OEE3 and LHCII-CAB proteins under LL conditions suggested that the plants tried to stabilize PSII and modulate their antenna size in order to capture more light and support photosynthesis. However, they were unable due to the down-regulation of OEE2, PsbP-like protein (PPL), and FNR. OEE2 is required for the assembly and/or stability of PSII and for the formation of PSII–LHCII super-complexes, whereas PPL is required for efficient repair of photodamaged PSII (Matsui et al., 2013). Recently, a proteomic analysis of *P. oceanica* collected from deep waters also revealed significant accumulation of LHC-CAB proteins and no change in OEE proteins, as compared to plants collected from shallow waters (Dattolo et al., 2013).

Only SSL conditions induced an up-regulation of proteins linked to photosynthesis such as FNR, and in proteins associated with energy production such as H^+^-ATPase and ATPase-β. This suggests that plants under SSL conditions have a high demand for energy (ATP) and reducing power (NADPH) to support reducing pathways such as carbon fixation and nitrogen metabolism (Ghosh and Xu, 2014; Komatsu et al., 2014). However, the down-regulation of FNR in LL conditions suggested a partial inhibition of electron transport, which impaired the production of reducing power and resulted in photoinhibition. Moreover, a significant reduction in the rETR_max_ of plants under LL conditions also supported the occurrence of photoinhibition. Our results are in contrast to those of Dattolo et al. (2013), who found that *P. oceanica* obtained from deep waters exhibited higher levels of FNR and ATPase-β compared to *P. oceanica* from shallow waters. An inadequate energy supply, or a reduced efficiency to generate energy, usually results in impairment of cellular metabolism and leads to senescence, which seems to be the case for plants grown under LL conditions.

Plants grown under SSL conditions had a significant increase in RuBisCO expression. This was apparently caused by the RuBisCO activase, which was also increased 3-fold. RuBisCO activase removes tightly bound sugar-phosphates from the active centers of RuBisCO (leading to its reactivation) and acts as a chaperone during stress. Its activity is known to be modulated by the redox status of the chloroplast stroma (Chen et al., 2015). The chloroplast-localized proteins PGK, GA3PDH, and transketolase (TK), that were accumulated under SSL conditions, are involved in the reduction and regeneration phases of the Calvin Benson cycle, and play a crucial role in maintaining the photosynthetic carbon flux during environmental stress conditions (Uberegui et al., 2015). Furthermore, their substrates and products act as precursors for associated metabolic processes such as amino acid and fatty acid synthesis. Therefore, variations in their abundance would affect other chloroplast pathways. The above results suggest that *Z. muelleri* can resist SSL stress through the up-regulation of proteins related to light-dependent reactions, which in turn provide adequate amounts of energy equivalents necessary for the Calvin cycle and other important metabolic processes. However, LL conditions induced a significant down-regulation of RuBisCO and other proteins of the Calvin cycle, suggesting a low intercellular CO_2_ concentration and a decrease in the rate of photosynthesis as a consequence. Consistent with this, RuBisCO has been reported to be down-regulated in the leaves of *P. oceanica* acclimated to chronic low-light conditions, which also exhibited reduced leaf growth and low protein yield (Mazzuca et al., 2009). On the contrary, RuBisCO and RuBisCO activase were up-regulated in *P. oceanica* collected from deep waters, as compared to *P. oceanica* from shallow waters (Dattolo et al., 2013). However, Dattolo et al. (2014) showed the up-regulation of the genes coding for the RuBisCO small subunit (*SSU5B*) and ferredoxin (*SEND33*) in plants collected from shallow waters, which supports our results. These contradictory findings suggest that genus-specific determinants underlie differences in the acclimation response, but fail to completely explain how RuBisCO is regulated in seagrasses grown under LL conditions, regardless of their genus.

### Remobilization of energy metabolism via glycolysis, Kreb's cycle, photorespiration, and amino acid metabolism

Plants facing altered environmental conditions generally need an enhanced supply of immediately available energy, which can be obtained from glycolysis (EMP) and the Krebs cycle (TCA). Several proteins from the EMP and TCA pathways were either up-regulated or newly appeared under SSL conditions, such as FBA, TPI, and NADP-GA3PDH. These are important enzymes of the EMP pathway and catalyze the three consecutive steps that convert fructose-1,6-bisphosphate into 1,3-bisphosphoglycerate, while generating NADPH (Figure 4). Moreover, accumulation of mtLPD (an important member of the pyruvate dehydrogenase complex and the glycine cleavage complex), aconitate hydratase, and citrate synthase under SSL conditions ensured the smooth functioning of the TCA cycle, by catalyzing the first three steps needed to convert pyruvate into isocitrate. Up-regulation of TCA and EMP enzymes in plants under SSL conditions contributed to glucose reduction, thus producing extra energy to cope with high light stress. Recent studies reveal a close connection between the abundance of EMP and TCA proteins and various abiotic stresses (Ghosh and Xu, 2014; Komatsu et al., 2014; Kosová et al., 2014). López-Cristoffanini et al. (2015) reported that up-regulation of TPI and AH provide the energy needed for protein synthesis in desiccated plants. The up-regulation of GA3PDH and TPI in *P. oceanica* under high light conditions further support the involvement of EMP proteins in energy balance during stress in seagrass (Dattolo et al., 2013).

Photorespiration is an important part of the stress response that helps minimize ROS production directly or indirectly using ATP and NADPH. Glyoxylate/hydroxypyruvate reductase B (HPR) is a peroxisomal photorespiratory enzyme that was up-regulated in *Z. muelleri* grown under SSL conditions. HPR converts hydroxypyruvate into glycerate, which later re-enters the C3 cycle for energy production. The proteins glycine dehydrogenase (GlyDH), aminomethyl transferase (AMT), and mtLPD were significantly accumulated in plants under SSL conditions (Figure 4). These proteins are essential components of the glycine cleavage complex (GCC), which together with GyHMT (formally recognized as SHMT) catalyze the conversion of glycine to serine, in a process that also generates ammonia, CO_2_, and NADH (Marchand et al., 2004). Furthermore, plants under SSL conditions exhibited the up-regulation of glutamate-ammonia ligase (also recognized as glutamine synthetase, GS), an enzyme that assimilates the ammonia generated in the GCC via the GS/GOGAT cycle by consuming reducing equivalents from ferrodoxin and/or NADPH/ATP. In turn, up-regulation of mtLPD, HPR, and GCC activity suggest that the plants under SSL conditions were regulating the photorespiratory carbon cycle, effectively shaping photosynthesis (Timm et al., 2015). This allows for increased performance of the C3 cycle and enhance the generation of ribulose-1,5-bisphosphate (RuBP), also facilitating the consumption of photorespiratory metabolites and, in turn, photosynthetic carbon assimilation (Voss et al., 2013). A significant accumulation of proteins associated with the GCC, together with SHMT upon light treatment, has also been reported in rice (Huang et al., 2013) and *Arabidopsis* (Lee et al., 2010).

Apparently, accumulation of glutamate dehydrogenase (GDH) and glutamate decarboxylase (GDC) in plants under SSL conditions suggests a coordinated effort to sustain nitrogen metabolism under stress. GDH and GDC together catalyzes the conversion of glutamate into γ-aminobutyric acid (GABA) (Figure 4), which act as a signal molecule to activate diverse metabolic pathways to combat stress in terrestrial and marine plants (Kumar et al., 2014, 2016; Hasler-Sheetal et al., 2015). Apart from these enzymes, a significant up-regulation of adenosylhomocysteinase (AHCY) and the new appearance of methionine synthase (MS), pyrroline-5-carboxylate reductase (P5CR), and inositol-3 phosphate synthase (IPS) suggests the involvement of organic osmolytes in ROS scavenging during SSL conditions. AHCY and MS are key enzymes in the synthesis of S-adenosylmethionine (SAM), which is an important precursor of several molecules that increase under stress, such as glycine betaine, polyamines, and ethylene (Figure 4; Kumar et al., 2014, 2015, 2016). Therefore, the up-regulation of the GCC together with enzymes of C3, TCA, and glutamate-proline-GABA pathways, suggest the fine-tuning of these interconnected pathways to control photosynthesis and growth of *Z. muelleri* under SSL conditions.

The down-regulation of proteins related to energy and amino acid metabolism in plants under LL conditions suggests insufficient energy for protein synthesis. This would impair C:N balance, which may in turn impair plant growth. Dattolo et al. (2013) also observed the down-regulation of cytosolic GA3PDH and PGK in *P. oceanica* grown under LL conditions. Interestingly, gamma carbonic anhydrase (γ-CA) was the only new protein that appeared under these conditions. This enzyme, which catalyzes the reversible conversion of ${\text{HCO}}_{3}^{-}$ to CO_2_, has been identified in the *Z. muelleri* transcriptome. In higher plants and photosynthetic algae, γ-CA enzymes are part of the respiratory complex 1 (NADH:ubiquinone oxidoreductase), and serve as the entry point of electrons to the mitochondrial respiratory electron transport chain (Figure 5), potentially playing a role in photorespiration (Brauna and Zabaleta, 2006).

### Antioxidant defense and related proteins that maintain the redox status

Stress causes an imbalance in the generation and scavenging of ROS, which in turn causes a disruption in cellular redox homeostasis. Marine plants express a battery of enzymatic and non-enzymatic antioxidants used to scavenge ROS and maintain cellular redox status (Kumar et al., 2014). We observed up-regulation or the new appearance of several antioxidant enzymes such as APX, CAT, POX, MDHAR, GSTs, and glutathione peroxidase (GPX), and of proteins involved in flavonoid synthesis such as IFR and DHFR. These results suggested that plant exposure to SSL conditions results in ROS production, which triggers a multi-enzyme antioxidant response especially in the ascorbate-glutathione cycle (AsA-GsH cycle). This cycle generates reducing antioxidants such as AsA and glutathione (GsH, an important non-enzymatic antioxidant that copes with various stresses), which help to maintain the redox status while scavenging H_2_O_2_ (Kumar et al., 2014). Proteins such as MPGT and GME, which were up-regulated under SSL conditions, participate in the synthesis of cell wall polysaccharides and are key enzymes in the ascorbate (AsA) biosynthesis pathway (Figure 4; Gilbert et al., 2009). Cystein synthase (CS), up-regulated under SSL conditions, not only is the precursor for SAM biosynthesis, but also catalyzes the rate-limiting step of GsH synthesis. Therefore, the appearance of CS in plants under SSL conditions reflects its role in relieving stress in multiple ways. Similarly, lactoylglutathione lyase, up-regulated under SSL conditions, is involved in the glutathione-based detoxification of methylglyoxal (MG), a toxic byproduct of carbohydrate, and amino acid metabolism. The accumulation of MG is indicative of abiotic stress conditions, such as desiccation in seaweeds (López-Cristoffanini et al., 2015).

Many secondary metabolites such as anthocyanins and flavonoids synthesized in the phenylpropanoid pathway are suggested to be crucial in osmotic and ROS scavenging in plants facing a wide range of environmental stressors (Petrussa et al., 2013). The high abundance of DFR (the first committed enzyme of anthocyanin biosynthesis) and IFR (involved in flavonoid synthesis) in plants under SSL conditions indicates a potential link between increased ROS levels and higher flavonoid and anthocyanin synthesis. Altogether, the differential regulation of proteins involved in the AsA-GsH cycle and flavonoid pathway indicates that SSL conditions triggered the plant antioxidant defense system, which improved the redox status and thus the SSL tolerance of *Z. muelleri*. Differential regulation of these antioxidant enzymes has been well documented as a defense response of seagrasses to light stress, heavy metal toxicity, and ocean acidification (Li et al., 2012; Dattolo et al., 2013; Lauritano et al., 2015). However, down-regulation of superoxide dismutase (SOD-Cu/Zn) and germin-like protein (that has both SOD and oxalate oxidase activity) suggests that this species is sensitive to high light stress. Surprisingly, it was found that none of the antioxidant enzymes were differentially regulated in plants under LL conditions, unlike Dattolo et al. (2013) and Mazzuca et al. (2009), who found CAT and SOD were up-regulated in *P. oceanica* grown under LL conditions.

### Proteostasis, trafficking, and others

Protein dysfunction is an inevitable consequence of a wide range of adverse environmental conditions, including light stress. Up-regulation of the chloroplast-localized elongation factor (EF-Tu) in plants under SSL conditions suggests enhanced synthesis of new proteins and prevention of the aggregation of degraded proteins, to better tolerate stress. EF-Tu has been suggested to act as a molecular chaperon for the RuBisCO activase, protecting it from aggregation due to heat stress (Ristic et al., 2007). Several heat-shock and/or chaperone proteins, including Hsp70, Hsp-STI1 (characterized as Hsp70/Hsp90 co-chaperones), chaperon-ClpB1, and chaperon-60 β were up-regulated in plants under SSL conditions. These proteins have been shown to participate in protein stabilization, folding, and assembly, preventing the aggregation of non-native proteins, thus assisting to fight abiotic stresses (Timperio et al., 2008). Consistent with our findings, several Hsps were up-regulated in *P. oceanica* obtained from shallow waters, as compared to *P. oceanica* from deep waters (Dattolo et al., 2013). Surprisingly, polyubiquitin 11 (that mediates ubiquitin proteasome-related protein degradation) and nascent polypeptide-associated complex subunit beta (NAC-β, protects nascent polypeptides from proteolysis) were down-regulated in plants under SSL conditions, their role in proteostasis for stress tolerance has been well documented in land plants (Kirstein-Miles et al., 2013). Therefore, Hsp/chaperones play a pivotal role in fighting SSL stress in *Z. muelleri*, by re-establishing proteostasis, and cellular homeostasis. In contrast, most proteostasis regulatory proteins (eEF-5A, NAC-β, and pTAC16), except for peptidyl-prolyl cis/trans isomerase (PPIase) (upregulated to 3 fold) were downregulated in plants under LL conditions. PPIase catalyzes *cis-trans* isomerization of the peptidyl-prolyl bond, which is a rate-limiting step in protein folding. This enzyme intervenes in protein folding in the marine seaweed *P. orbicularis* when exposed to desiccation (López-Cristoffanini et al., 2015), which supports its protective role under stress conditions. However, down-regulation of most proteostasis proteins under LL conditions suggests a premature senescence in these plants (Pang et al., 2010), which could be related to the lower rETR_max_ value observed in plants under LL conditions, as compared to plants under control conditions.

The alpha-soluble NSF attachment protein (αSNAP) was enhanced in both SSL and LL conditions, in contrast to the ADP-ribosylation factor (ARF). The SNAPs and ARFs are essential in vesicle trafficking, and it has been shown that SNAPs coupled with SNAREs mediate ROS delivery to vacuoles through endosomal vesicle fusion, to fight osmotic stress in *Arabidopsis* (Leshem et al., 2006). These results suggested that *Z. muelleri* grown under SSL or LL conditions uses a similar mechanism for targeting ROS to subcellular compartments. Further, nucleoside diphosphate kinase (NDPK) and adenylate kinase (ADK) are well known for maintaining the cellular nucleotide pool; however, they were recently demonstrated to have a role in the ROS signaling and detoxification processes while interacting with CAT, G proteins, and MAP kinases (Yoshida et al., 2006). The down-regulation of these proteins at least in LL conditions suggests a negative impact of LL stress on the nucleotide pool synthesis and GTP-mediated signaling pathways.

## Conclusion

*Z. muelleri* grown under SSL conditions had a large number of up-regulated and new proteins that appeared, compared to *Z. muelleri* grown under LL conditions. This suggests that seagrasses make use of a genetic plasticity to cope with stress induced by SSL. Overall, the proteomic analysis revealed the physiological tolerance of *Z. muelleri* to SSL stress, given its ability to modulate primary and secondary metabolism. Figure 4 summarizes the mechanisms proposed to explain *Z. muelleri* response to SSL stress. In contrast, LL conditions induced the down-regulation of key metabolic enzymes of photosynthesis, carbohydrate and amino acid metabolism, and proteostasis, to negatively affect the metabolic activities of *Z. muelleri* and to reduce photosynthetic performance (Figure 5). Growth under SSL conditions induced the accumulation of proteins involved in cell wall hardening and osmoregulation (which were up-regulated under hypersalinity stress in the seagrass *C. nodosa*; Piro et al., 2015); however, further exploration is needed to determine if the strategies to cope with light and salinity stress are similar. The proteomic profile developed in this study, and the knowledge we obtained from it, could serve as a basis for future system biology research in seagrasses, in order to fully understand their response to global climate change.

## Author contributions

MK, MP, PD and PR conceived and designed research. MK, MPP performed 2D-IEF and protein identification using LC-MS/MS and analyzed the data. PD and MP determined photosynthetic performance in field and in laboratory based experiments. MK, MPP, PD, MP and PR wrote manuscript. ZJ assisted in protein sample preparation for LC-MS/MS and GS generated ESTs based peptide sequence and assisted in bioinformatics analytical tools. PR and LC revised the paper. All authors read and approved the manuscript.

### Conflict of interest statement

The authors declare that the research was conducted in the absence of any commercial or financial relationships that could be construed as a potential conflict of interest.

## Supplementary material

Supplementary Figure 1 **Rapid Light Curve measurements for *Zostera muelleri* seagrass plants exposed to saturating light (SL Control; 200 μmol photons m^−2^ = s^−1^), super-saturating light (SSL, 600 μmol photons m^−2^s^−1^), and limited light (LL, 20 μmol photons m^−2^s^−1^) irradiance at (A)** day 0 (T0) and **(B)** day 10 (T10) of the experiment.

Supplementary Figure 2 **Relative volume variation of *Zostera muelleri* protein spots between super-saturating light (SSL; 600 μmol photons m^−2^s^−1^) and saturating light (SL, Control; 200 μmol photons m^−2^s^−1^) irradiance conditions**. Numbers in brackets correspond to the spot number of each identified protein as given in Table 1. The relative protein spot volume variation in all the differential spots between both conditions was significantly different (One-Way ANOVA; *p* ≤ 0.05). ECBM, energy carbohydrate and biomolecule metabolism; PS, photosynthesis; SM, secondary metabolism; SVT, signaling and vesicle trafficking; ADS, antioxidant defense system; GIP, genetic information processing; O, others.

Supplementary Figure 3 **Relative volume variation of *Zostera muelleri* protein spots between limited light (LL; 20 μmol photons m^−2^s^−1^) and saturating light (SL, Control; 200 μmol photons ms^−1^) irradiance conditions**. Numbers in brackets correspond to the spot number of each identified protein as given in Table 2. The relative protein spot volume variation in all the differential spots between both conditions was significantly different (One- Way ANOVA; *p* ≤ 0.05). Refer Supplementary Figure 2 for extended form of abbreviated functional categories.

Supplementary Table 1 **List of proteins identified, their peptide sequences and associated statistics for (Sheet A) super-saturating light (SSL; 600 μmol photons m^−2^s^−1^) and saturating light (SL, Control; 200 μmol photons m^−2^s^−1^) irradiance conditions; and for (Sheet B) limited light (LL; 20 μmol photons m^−2^s^−1^) and saturating light (SL, Control; 200 μmol photons ms^−1^) irradiance conditions. Protein name with spot numer, accession number, total peptide-spectrum matches (among them unique peptides are represented with Y) found for each protein, peptide molecular weight, their retention time, m/z value, −10lgP and other statistical parameters are shown**.
