# Supplementary figures and images for: Proteome Analysis Reveals Extensive Light Stress-Response Reprogramming in the Seagrass Zostera muelleri (Alismatales, Zosteraceae) Metabolism
Source: Front Plant Sci. 2017 Jan 17;7:2023. doi: 10.3389/fpls.2016.02023 (PMC5239797; doi:10.3389/fpls.2016.02023)

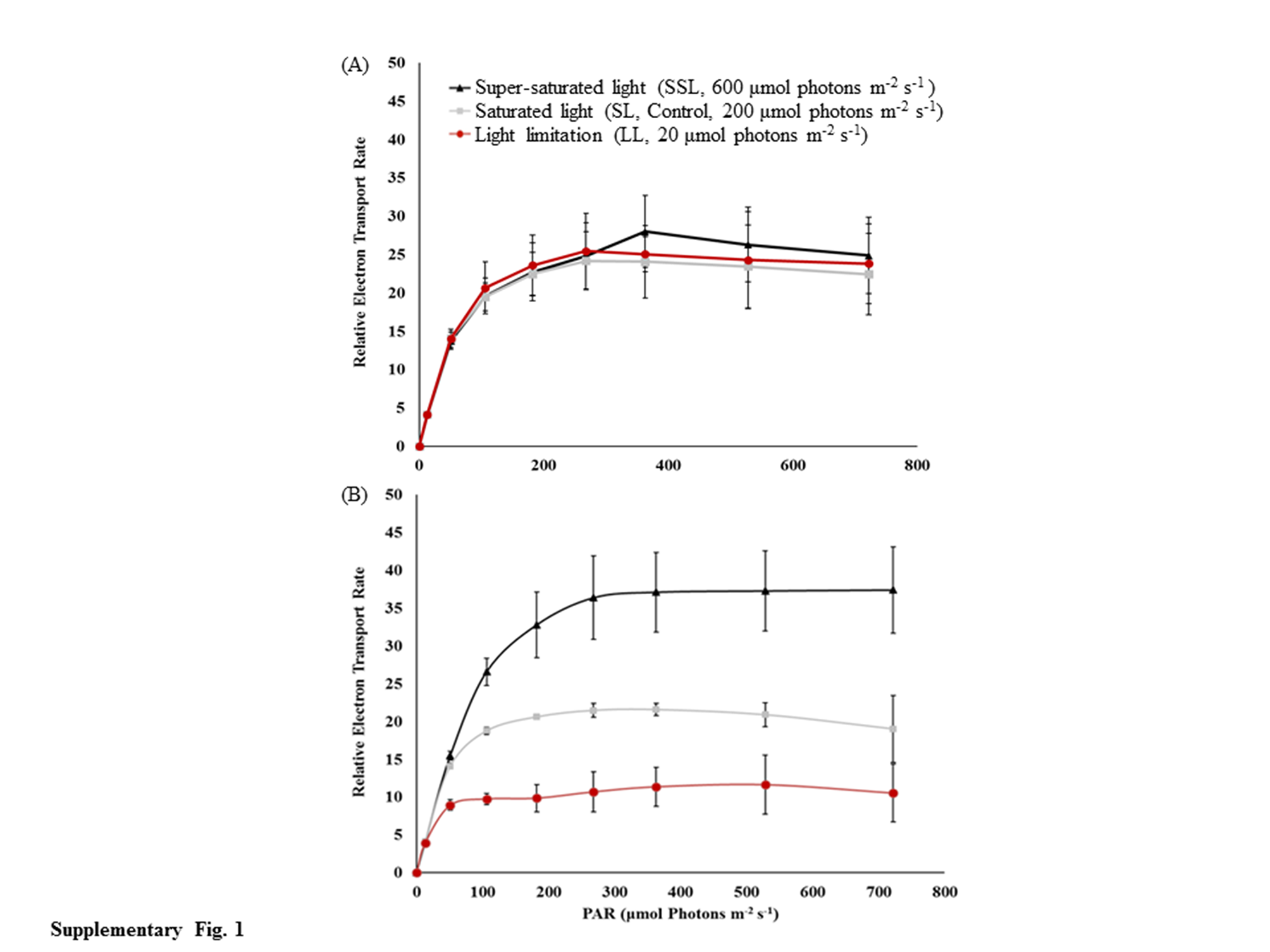

Supplement: Supplementary Figure 1 — Rapid Light Curve measurements for Zostera muelleri seagrass plants exposed to saturating light (SL Control; 200 μmol photons m−2 = s−1), super-saturating light (SSL, 600 μmol photons m−2s−1), and limited light (LL, 20 μmol photons m−2s−1) irradiance at (A) day 0 (T0) and (B) day 10 (T10) of the experiment. [file Image1.TIF]

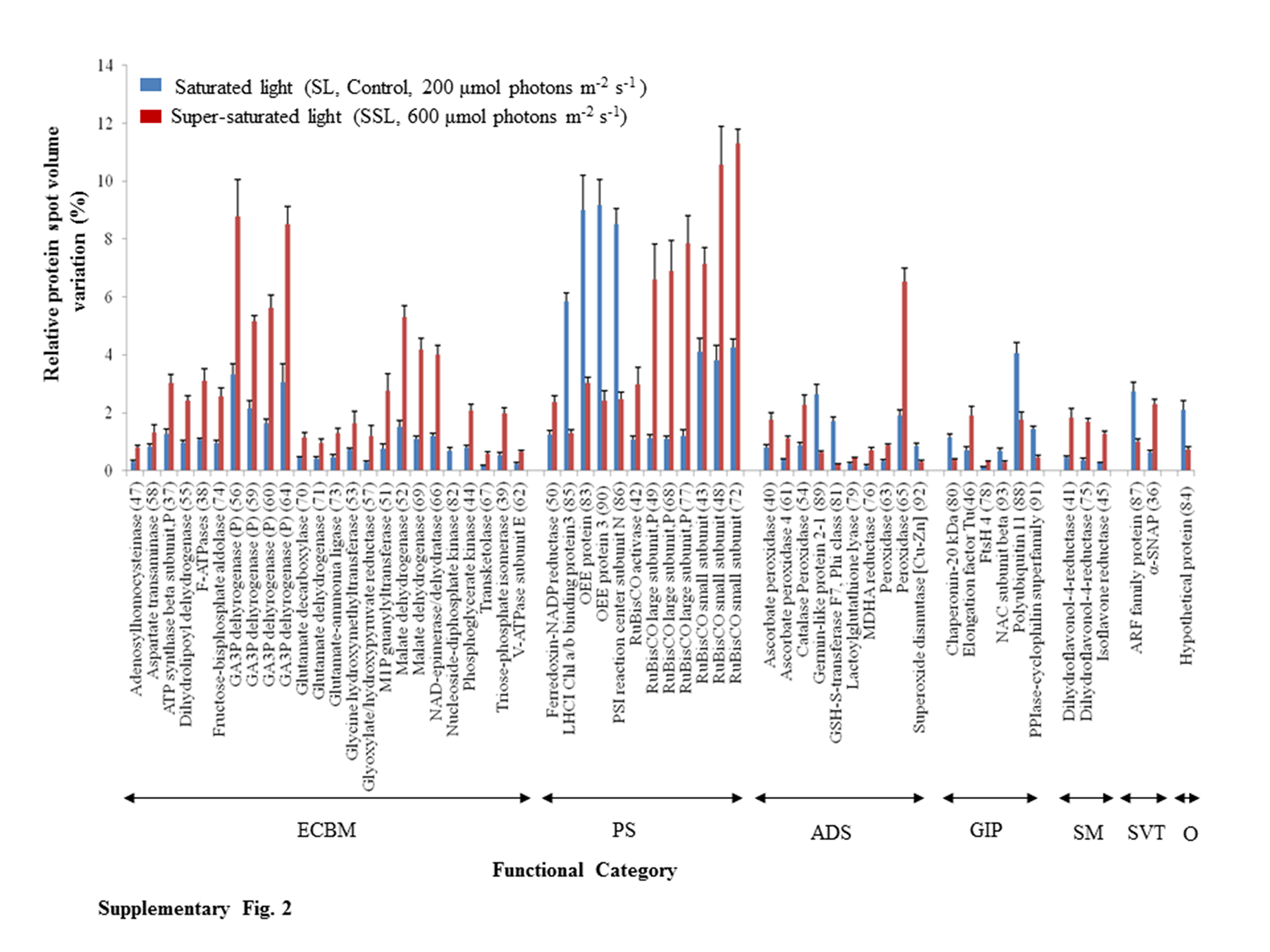

Supplement: Supplementary Figure 2 — Relative volume variation of Zostera muelleri protein spots between super-saturating light (SSL; 600 μmol photons m−2s−1) and saturating light (SL, Control; 200 μmol photons m−2s−1) irradiance conditions. Numbers in brackets correspond to the spot number of each identified protein as given in Table 1. The relative protein spot volume variation in all the differential spots between both conditions was significantly different (One-Way ANOVA; p ≤ 0.05). ECBM, energy carbohydrate and biomolecule metabolism; PS, photosynthesis; SM, secondary metabolism; SVT, signaling and vesicle trafficking; ADS, antioxidant defense system; GIP, genetic information processing; O, others. [file Image2.TIF]

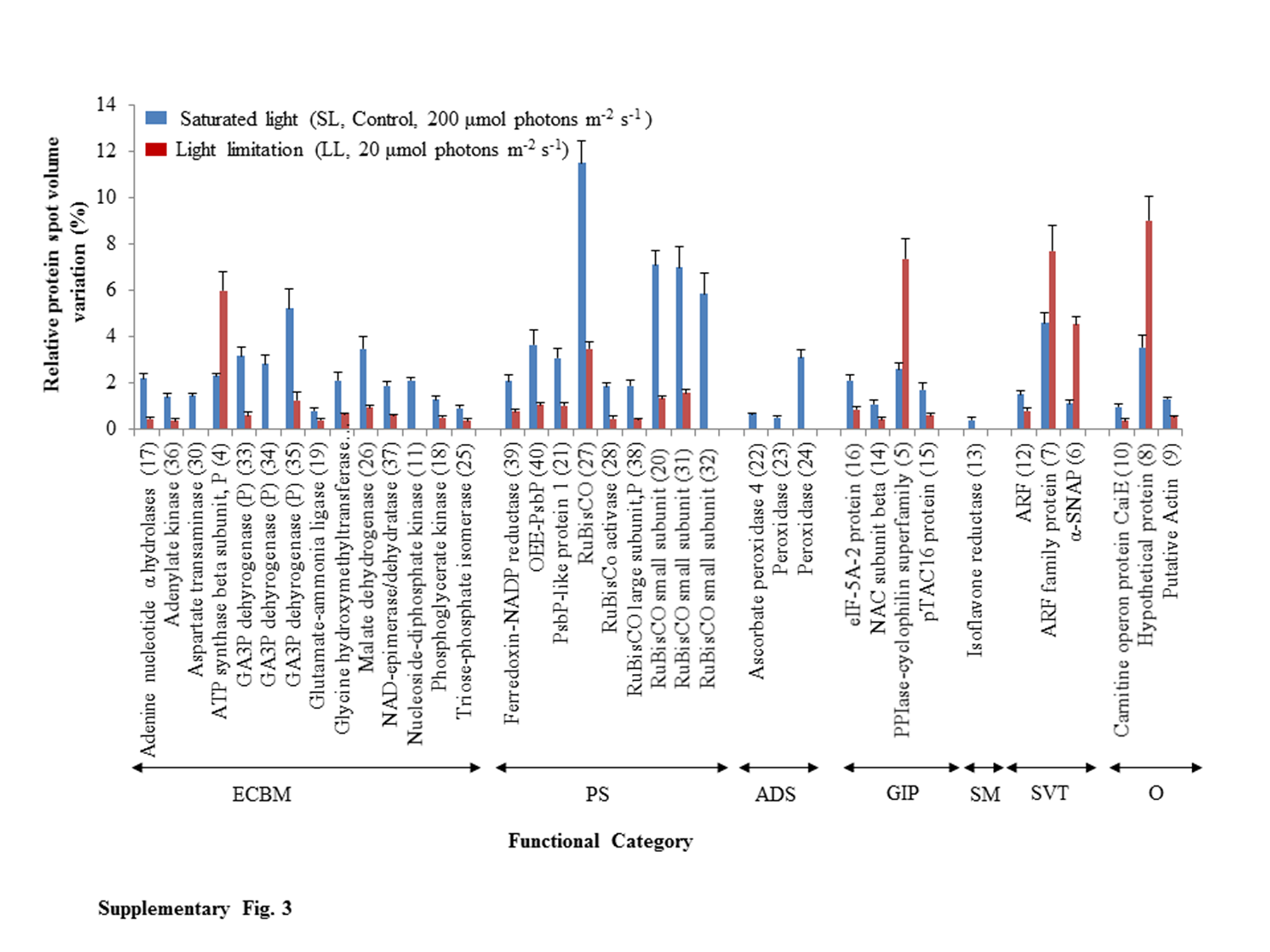

Supplement: Supplementary Figure 3 — Relative volume variation of Zostera muelleri protein spots between limited light (LL; 20 μmol photons m−2s−1) and saturating light (SL, Control; 200 μmol photons ms−1) irradiance conditions. Numbers in brackets correspond to the spot number of each identified protein as given in Table 2. The relative protein spot volume variation in all the differential spots between both conditions was significantly different (One- Way ANOVA; p ≤ 0.05). Refer Supplementary Figure 2 for extended form of abbreviated functional categories. [file Image3.TIF]
